# Peritumoral administration of DRibbles-pulsed antigen-presenting cells enhances the antitumor efficacy of anti-GITR and anti-PD-1 antibodies via an antigen presenting independent mechanism

**DOI:** 10.1186/s40425-019-0786-7

**Published:** 2019-11-20

**Authors:** Jaina M. Patel, Zhihua Cui, Zhi-Fa Wen, Catherine T. Dinh, Hong-Ming Hu

**Affiliations:** 1 Laboratory of Cancer Immunobiology, Robert W. Franz Cancer Research Center, Earle A. Chiles Research Institute, Providence Cancer Center, 4805 NE Glisan Street, Portland, OR 97213 USA; 2 Department of Microbiology and Immunology, Medical School of Southeast University, Nanjing, Jiangsu Province People’s Republic of China

**Keywords:** GITR, PD-1, Antigen presenting cells, Dendritic cells, Peritumoral injection, Tumor microenvironment

## Abstract

**Background:**

TNF receptor family agonists and checkpoint blockade combination therapies lead to minimal tumor clearance of poorly immunogenic tumors. Therefore, a need to enhance the efficacy of this combination therapy arises. Antigen-presenting cells (APCs) present antigen to T cells and steer the immune response through chemokine and cytokine secretion. DRibbles (DR) are tumor-derived autophagosomes containing tumor antigens and innate inflammatory adjuvants.

**Methods:**

Using preclinical murine lung and pancreatic cancer models, we assessed the triple combination therapy of GITR agonist and PD-1 blocking antibodies with peritumoral injections of DRibbles-pulsed-bone marrow cells (BMCs), which consisted mainly of APCs, or CD103+ cross-presenting dendritic cells (DCs). Immune responses were assessed by flow cytometry. FTY720 was used to prevent T-cell egress from lymph nodes to assess lymph node involvement, and MHC-mismatched-BMCs were used to assess the necessity of antigen presentation by the peritumorally-injected DR-APCs.

**Results:**

Tritherapy increased survival and cures in tumor-bearing mice compared to combined antibody therapy or peritumoral DR-BMCs alone. Peritumorally-injected BMCs remained within the tumor for at least 14 days and tritherapy efficacy was dependent on both CD4+ and CD8+ T cells. Although the overall percent of tumor-infiltrating T cells remained similar, tritherapy increased the ratio of effector CD4+ T cells-to-regulatory T cells, CD4+ T-cell cytokine production and proliferation, and CD8+ T-cell cytolytic activity in the tumor. Despite tritherapy-induced T-cell activation and cytolytic activity in lymph nodes, this T-cell activation was not required for tumor regression and enhanced survival. Replacement of DR-BMCs with DR-pulsed-DCs in the tritherapy led to similar antitumor effects, whereas replacement with DRibbles was less effective but delayed tumor growth. Interestingly, peritumoral administration of DR-pulsed MHC-mismatched-APCs in the tritherapy led to similar antitumor effects as MHC-matched-APCs, indicating that the observed enhanced antitumor effect was mediated independently of antigen presentation by the administered APCs.

**Conclusions:**

Overall, these results demonstrate that peritumoral DR-pulsed-BMC/DC administration synergizes with GITR agonist and PD-1 blockade to locally modulate and sustain tumor effector T-cell responses independently of T cell priming and perhaps through innate inflammatory modulations mediated by the DRibbles adjuvant. We offer a unique approach to modify the tumor microenvironment to benefit T-cell-targeted immunotherapies.

## Background

Peripheral administration of checkpoint inhibitors against PD-1 and CTLA-4 are beneficial against a subset of patients of most cancer types, yet fail to show responses in all patients, primarily due to low tumor mutation burden and pre-existing immunity. To further boost antitumor T-cell responses, multiple combination strategies have been tested in preclinical animal models and clinical trials. One method combines agonist antibodies against TNF receptor (TNFR) family members with checkpoint blockade [1–4], such as targeting GITR and blocking PD-1 together. GITR agonist increases activation, proliferation and effector function of CD8+ and CD4+ T cells [5–7], while decreasing intra-tumor regulatory T cells (Tregs) by depletion [8, 9] and Treg lineage stability alterations [10, 11], thus proving effective in various preclinical tumor models [7, 12, 13]. Recent studies combining anti-GITR and anti-PD-1 antibodies led to the rescue of dysfunctional/exhausted CD8+ T cells [14, 15], and increased tumor infiltration of effector and memory T cells with decreased Tregs and myeloid derived suppressor cells (MDSCs) [2, 4, 16]. Although combined anti-GITR and anti-PD-1 antibody therapy delayed tumor growth in murine tumor models compared to single antibody administration, minimal clearance of tumors was detected without using an additional immune activating component, such as chemotherapy, vaccination or radiation, early during treatment [2, 4, 16]. This minimal clearance was presumably due to the inadequate ability of tumor-infiltrating T cells to expand and sustain effector function against local immune suppression within the tumor. Although chemotherapy and radiation therapy increases tumor antigenicity and removes immunosuppressive cells from the tumor microenvironment (TME) [17], toxic side effects arise. A safer method to modulate the immunosuppressive TME to an immune-stimulating one that sustains T-cell function will prove to be beneficial.

Antigen presenting cells (APCs) present antigen, provide costimulation, and secrete chemokines/cytokines to steer and control the direction of the immune response. Direct peritumoral/intratumoral dendritic cell (DC) injections are more beneficial than subcutaneous administration [18], due to increased pro-immune cytokine production and tumor CD8+ T-cell infiltration, along with decreased Treg infiltration, tumor cell proliferation via TNF-α [19] and immunosuppressive cytokines [20]. DRibbles are tumor-derived autophagosomes that contain tumor proteins and peptides [21–23]. Long-lived peptides, usually degraded by lysosomes, and short-lived peptides that are quickly ubiquitinated and degraded by proteasomes are both present within DRibbles [21, 24]. In addition, DRibbles contain many damage associated molecular patterns (DAMPs) that act as danger signals and induce innate inflammatory responses [21, 23, 25]. Therefore, upon uptake by APCs, DRibbles can provide antigen as well as inflammatory danger signals. Given the robust peripheral immune activation but lack of sustained tumor effector T cells seen with TNFR agonist and checkpoint blockade, we hypothesize that GITR agonist and PD-1 blockade antibody therapy can be benefited by the additional administration of peritumoral DRibbles-pulsed-APCs that can modulate the local TME towards an immune-stimulating environment.

Home to both the common myeloid and lymphoid progenitor cells, the bone marrow gives rise to a variety of immune cells, including APCs. Herein, we present that the efficacy of systemically administered GITR agonist and PD-1 blockade is enhanced by peritumoral delivery of DRibbles-pulsed-bone marrow cells (BMCs) or DCs. This study proposes that peritumoral DR-pulsed-APC delivery following systemic T-cell targeted therapies, can sensitize the local TME to create a supportive environment that sustains T-cell immunity, independently of antigen presentation and perhaps through local inflammatory modulations.

## Materials and methods

### Mice

6–8 week old BALB/c or C57BL/6 mice were purchased from Jackson Laboratories. All experiments were conducted in accordance with Earle A. Chiles Research Institute (EACRI) approved Institutional Animal Care and Use Committee (IACUC) protocols.

### DRibbles preparation

DRibbles were prepared as previously described [26]. Line-1 or Panc02 tumor cell lines were treated with 100 nmol/L bortezomib and 10 mmol/L NH_4_Cl for 18 h. Autophagosomes were released via vigorous pipetting in wash buffer (PBS 5 mM EDTA, 20 mM NH_4_Cl), and centrifuged at 1000 rpm for 7 min. Supernatant containing DRibbles was washed three times by centrifuging at 7500 rpm for 15 min at 4 °C. The resulting DRibbles pellet was aliquoted in 6% hetastarch and stored at − 80 °C until use.

### Cells and antibodies

Line-1 cells, a gift from Dr. Anderson (University of Louisville School of Medicine, Microbiology and Immunology), was derived from a spontaneous BALB/c lung tumor [27]. Received Line-1 cells were passaged through a BALB/c mouse. The subcutaneous tumor was harvested, cultured for 4 days, and aliquots were frozen. Murine Panc02 and Panc02-SIY pancreatic cancer cells (gifted by Dr. Gough, EACRI) were thawed and expanded to generate a large cell bank. All cells were cultured in RPMI 1640, 10% fetal bovine serum (FBS) and 50 μg/ml gentamicin. For each experiment, frozen cell aliquots were thawed and cultured for 2–3 days before tumor inoculation.

Bone marrow cells were isolated from femurs and tibias of naïve mice. Red blood cells were lysed using ACK lysis buffer (Life Technologies) and cells were plated in petri dishes at a concentration of 2 × 10^6^ cells/ml in complete media (CM; RPMI 1640, 10% FBS, 50 μg/ml gentamicin, 1:1000 β-mercaptoethanol) for 8–9 days. Dendritic cells were generated from bone marrow cells as described previously [28].

The agonist anti-GITR antibody (Clone DTA-1 – gifted by Dr. S. Sakaguchi, Kyoto University, Kyoto, Japan), anti-PD-1 antibody (Clone G4 – a gifted by Dr. C. Drake, Johns Hopkins University), anti-CD4 antibody (Clone GK1.5) and anti-CD8 antibody (Clone YTS 169.4) were purified from hybridoma supernatant using a Protein G affinity column. Antibody endotoxin levels were tested using ToxinSensor™ Chromogenic LAL (GenScript) to ensure low levels.

### Tumor challenge and treatment

BALB/c and C57BL/6 mice were injected subcutaneously with 2 × 10^5^ Line-1 or Panc02 cells, respectively, by the right hind leg. 200 μg rat-anti-GITR antibody (Ab) was intraperitoneally (i.p.) administered on day 5 and 8. 200 μg hamster-anti-PD-1 Ab was i.p administered on days 10, 12 and 14. On day 12, 2 × 10^6^ DR-pulsed BMCs or DCs was injected peritumorally (p.t.) in 40 μL PBS. Mice were randomized before starting antibody injections and antibody-treated mice were randomized before p.t. injections. Mice with tumors greater than 150 mm^2^ were sacrificed according to IACUC guidelines.

### Flow cytometry analysis of tissue infiltrating cells

Tumors, lymph nodes (LNs) and spleens were mechanically dissociated. RBCs were lysed from splenocytes using ACK lysis buffer. Minced tumors were shaken at 37 °C in CM containing 1 mg/ml Collagenase IV (Worthington Biochemical) and 10 μg/ml F68 for 1 h and dissociated using a GentleMACS Dissociator. Flow cytometry staining was performed on single cell suspensions (Additional file 1: Table S1). Samples were run on either BD LSRII or BD LSRFortessa.

#### Statistical analysis

GraphPad Prism 7.01 was used to perform statistical tests. Kaplan-Meier survival curves were assessed using the Log-rank Mantel-Cox test. One-way ANOVA with Tukey’s multiple comparisons test or an unpaired Student’s t-test was used with data represented as Mean ± SD: * = *p* < 0.5, ** = *p* < 0.01, *** = *p* < 0.001, **** = *p* < 0.0001.

## Results

### The efficacy of systemic agonist anti-GITR antibody and PD-1 blockade is enhanced by local peritumoral DR-BMC administration

**Fig. 1:**
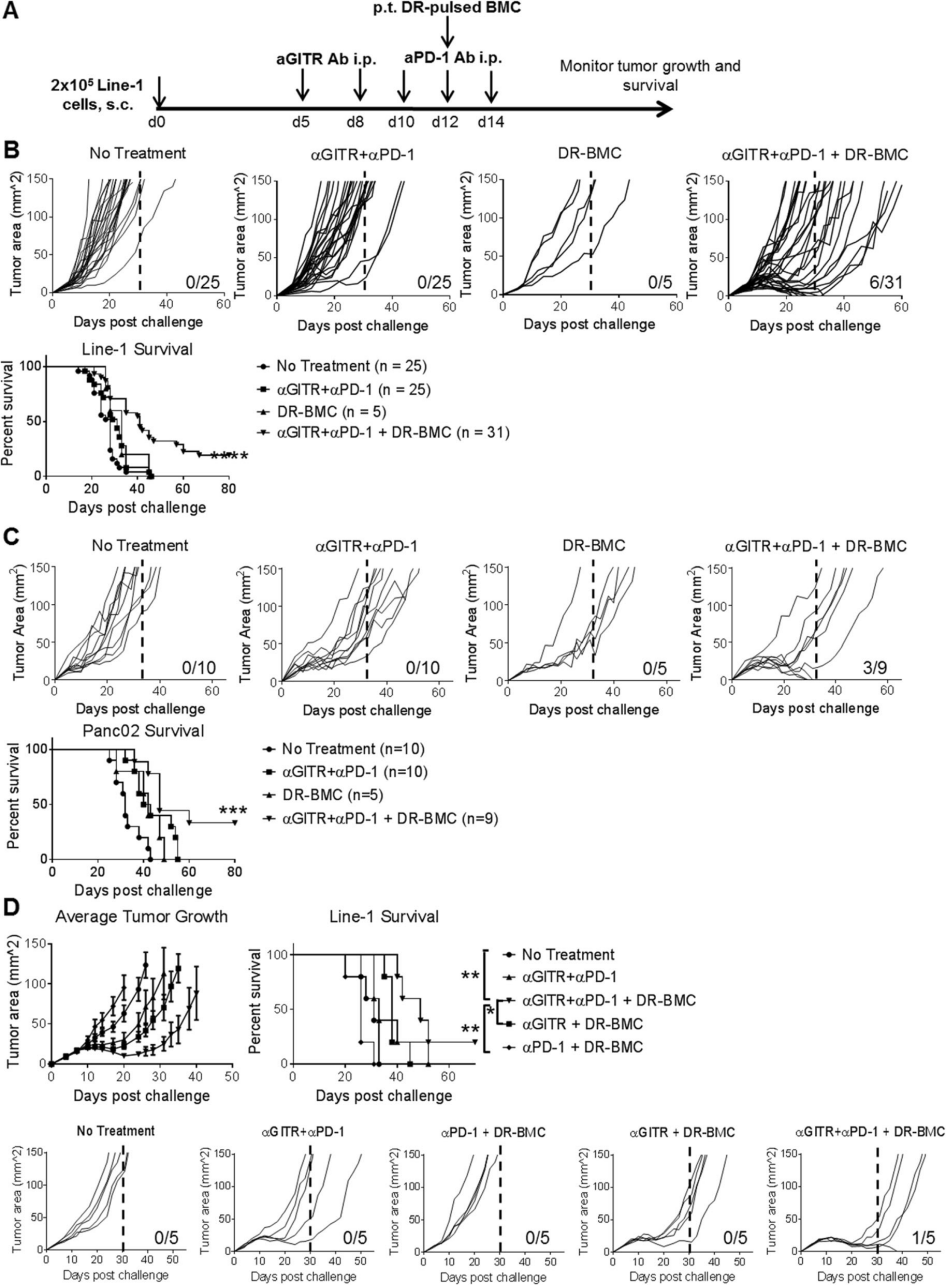
Peritumoral BMC vaccination enhances the survival of GITR agonist and PD-1 blockade treated tumor-bearing mice. **a**, Experimental schematic. **b**, Line-1-tumor bearing mice individual tumor growth curves and overall survival. Pooled data from 5 independent experiments are shown. **c**, Panc02-tumor bearing mice individual tumor growth curves and overall survival. Pooled data from 2 independent experiments are shown. **d**, Line-1 tumor bearing mice individual tumor growth curves and overall survival. Representative data from 2 independent experiments are shown (*n* = 5)

To assess if peritumoral administration of BMCs enhances the efficacy of systemically administered GITR agonist and PD-1 blockade, the poorly immunogenic lung cancer cell line, Line-1, was used. Checkpoint blockade following TNFR agonists enhances tumor clearance compared to simultaneous administration of both antibodies [3], therefore, we administered GITR agonist Ab first followed by PD-1 Ab blockade in Line-1 tumor-bearing BALB/c mice (Fig. 1a). BMCs were pulsed with the DRibbles vaccine derived from Line-1 tumor cells before administration to provide antigen and further activate APCs. Mice treated with antibody therapy (anti-GITR and anti-PD-1 Abs) or peritumoral DR-BMCs showed a modest delay in tumor growth with a median survival of 31 and 33 days, respectively, compared to untreated mice (median survival – 28 days). However, all mice succumbed to tumor outgrowth (Fig. 1b). In contrast, mice treated with the tritherapy (anti-GITR, anti-PD-1 and peritumoral BMCs) demonstrated the best efficacy with tumor growth further delayed (median survival of 41 days) and 19.3% complete tumor regression. Interestingly, the delayed survival in approximately 32% of the mice treated with tritherapy was due to tumors that began to regress but ultimately relapsed and continued to grow. Similar results were seen when using the poorly immunogenic Panc02 pancreatic cancer model in C57BL/6 mice by which 33.3% of the tritherapy-treated mice were cured with a median survival of 47 days, as compared to no cures seen in the untreated (median survival – 32 days), antibody treated (median survival – 41.5 days) or DR-BMC treated mice (median survival – 42 days) (Fig. 1c). Interestingly, administration of DR-BMCs earlier during the antibody treatment regimen, at day 8 or day 10, advanced median survival to 32 days compared to 42 days seen when DR-BMCs were administered at day 12 (Additional file 1: Figure S1). These results demonstrated that peritumoral DR-BMC administration enhanced the efficacy of systemically administered GITR agonist and PD-1 blockade, especially when given delayed at day 12.

The necessity of each individual antibody with peritumoral DR-BMCs was assessed (Fig. 1d). No tumor growth benefit was seen with PD-1 blockade and peritumoral DR-BMCs compared to untreated mice. Mice receiving GITR agonist with peritumoral DR-BMCs or PD-1 blockade showed delayed tumor growth kinetics but no cures, suggesting an important yet insufficient role of GITR agonist in generating a robust antitumor response. However, tritherapy-receiving mice experienced prolonged survival with a 20% cure rate. Therefore, the combination of all three components, GITR agonist, PD-1 blockade and peritumoral DR-BMCs, led to delayed tumor growth and increased survival.

### Peritumoral BMCs remain in the tumor for at least 2 weeks

Before peritumoral administration, BMCs expressed varying levels of MHC II, CD11c and CD11b (Additional file 1: Figure S2). Most cells expressed the DC marker, CD24, and a small population expressed the macrophage marker, F4/80. Very low Clec9a expression was detected and only a small population of MHC II+ cells expressed CD103 and IRF8, markers for cross-presenting DCs. A substantial population (~ 20%) did express GR1, commonly found on neutrophils and MDSCs.

**Fig. 2:**
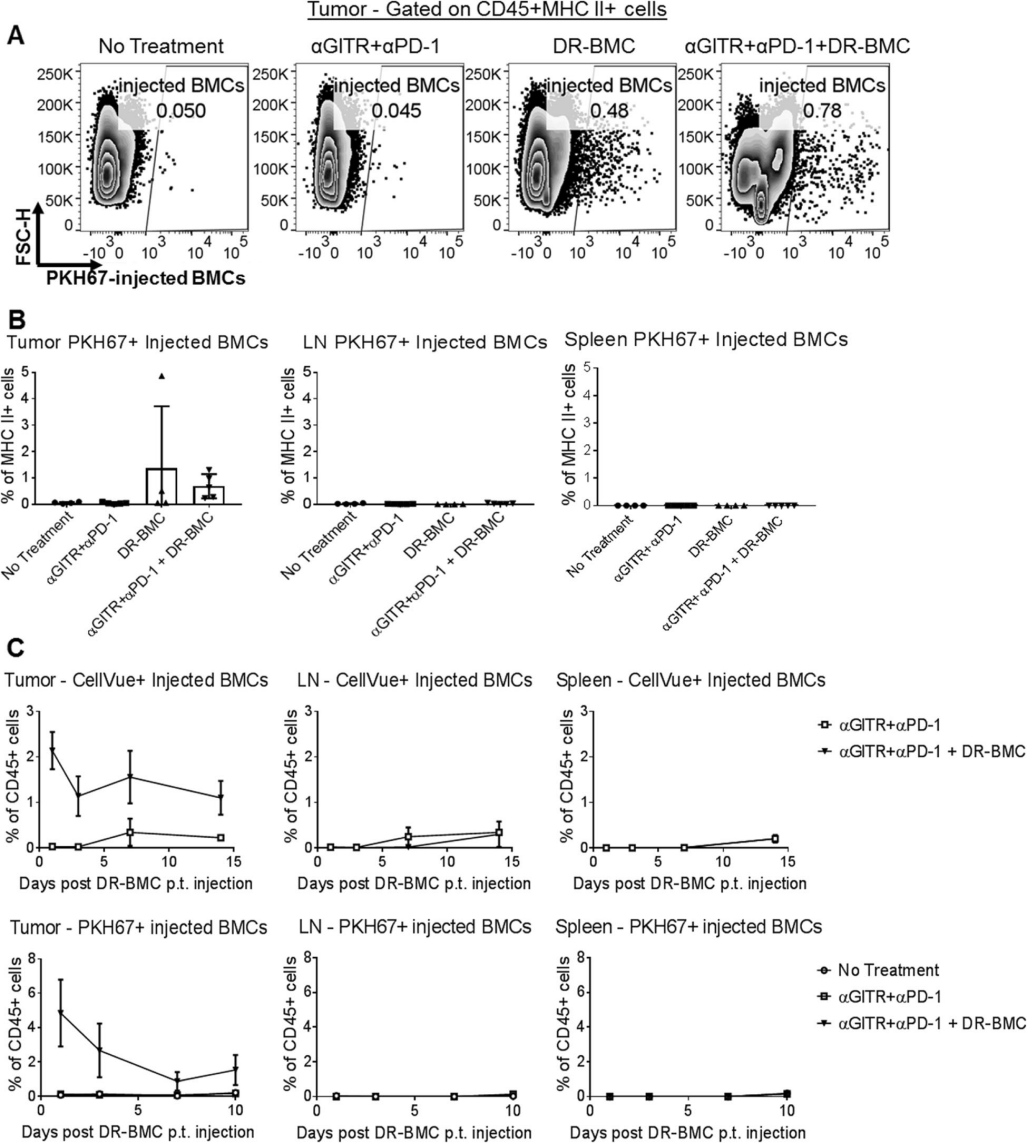
Peritumorally injected BMCs remain in the tumor of tritherapy-treated mice. **a**, Treated mice were euthanized 7 days after peritumoral injection of PKH67-labeled BMCs. Representative dot plots of PKH67-labeled cells from tumors of one experiment (n = 5) are shown. **b**, PKH67-labeled cells in the tumor, LN or spleen of mice 7 days after peritumoral vaccination of PKH67-labeled BMCs. Representative data (mean ± SD) of 5 mice in the antibody and tritherapy group and of 4 mice in the untreated and BMC only group from one independent experiment is shown. **c**, (Top row) Percent of CellVue+ cells found in the tumors, LNs and spleens of antibody treated mice injected with or without peritumoral CellVue-labeled BMCs 1, 3, 7 or 14 days after peritumoral BMC vaccination. Data represents the mean ± SEM of 3 mice in the tritherapy group and mean ± SEM of 2 mice in the antibody therapy group from one experiment. (Bottom row) Percent of PKH67+ cells found in the tumors, LNs and spleens of antibody treated mice injected with or without peritumoral PKH67-labeled BMC 1, 3, 7 or 10 days after peritumoral BMC vaccination. The data represents the mean ± SEM of 3 mice in the antibody and tritherapy group and 5 mice in the untreated group on days 1,3, and 10 whereas on day 7, mean ± SEM of 4 mice for each group is displayed from one experiment

Previous reports show that intratumorally-injected DCs labeled with a lipophilic dye trafficked to draining LNs [29]. We assessed trafficking patterns of peritumorally-injected DR-BMCs during tritherapy. DR-pulsed-BMCs were labeled with a lipophilic dye (CellVue Claret or PKH67) before peritumoral injections. Flow cytometry analysis of tumors, LNs and spleens harvested 7 days after DR-BMC administration demonstrated that the BMCs remained in the tumor at this time point and were not detected in the LN or spleen (Fig. 2a-b). A time-course study showed live dye-labeled BMCs present in the tumor for at least 14 days after peritumoral injections but still undetectable in the LNs or spleens (Fig. 2c). Injected BMCs expressed similar levels of MHC II, CD11c and CD11b 7 days after peritumoral injections as they did before injections, with low or undetectable IRF8 and CD103 expression (Additional file 1: Figure S3), suggesting that the lipophilic dye identified BMCs after tumor administration and that the TME did not affect expression of these molecules. Additionally, ~ 28% of the injected BMCs expressed the LN homing receptor, CCR7, although BMCs were undetectable in the LNs by flow cytometry. Approximately 30% of the BMCs demonstrated proliferation by Ki-67 expression and approximately 40% of the BMCs expressed the inhibitory molecule PD-L1. Therefore, peritumorally-injected DR-BMCs remained locally within the tumor for at least 2 weeks post administration and some were capable of proliferating inside tumors.

### Efficacy of Tritherapy depends on CD8+ and CD4+ T cells

To determine if a memory immune response was generated by the tritherapy, tritherapy-treated mice in which Line-1 or Panc02 tumors completely regressed were rechallenged with Line-1 or Panc02 tumor cells, respectively, on the opposite flank. In the Line-1 model, 80% (4 out of 5) of the rechallenged mice remained tumor-free whereas tumors grew out in all control mice (Additional file 1: Figure S4A). The Line-1 rechallenged mouse that did grow a tumor had delayed tumor kinetics in which a palpable tumor was not detected until 20 days after rechallenge as opposed to 5–7 days seen in the control mice. In the Panc02 model, 100% of the rechallenged mice remained tumor-free (Additional file 1: Figure S4B).

**Fig. 3:**
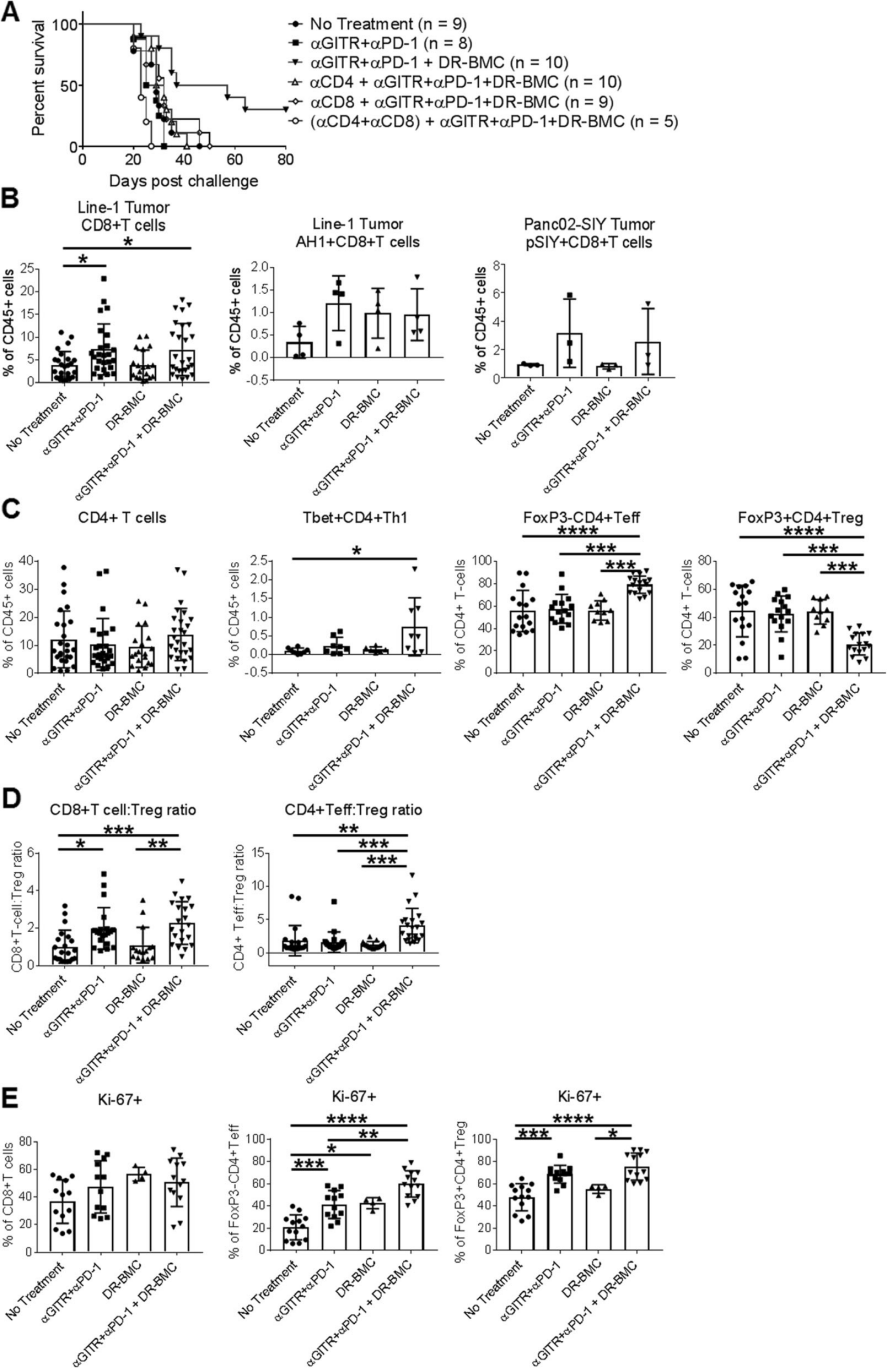
CD8+ and CD4+ T cells are required for tritherapy efficacy. **a**, Tritherapy-treated mice were depleted of CD4+ and/or CD8+ cells 1 day before beginning anti-GITR antibody administration. Survival was assessed. Pooled data from 2 independent experiments is shown. **b**, Tumors from line-1-tumor bearing mice were harvested 7 days after p.t. DR-BMC injections and analyzed by flow cytometry for total CD8+ T-cell (left) and AH1-tetramer-specific CD8+ T-cell (middle) infiltration into the tumor. Tumors from Panc02-SIY tumor bearing mice were harvested 10 days after p.t. DC vaccination and analyzed by flow cytometry for SIY-specific CD8+ T cells (right). Line-1 tumor CD8+ T-cell data demonstrates pooled data from 6 independent experiments whereas mean ± SEM from one independent experiment each is shown for AH1 + CD8+ T cells (*n* = 4) and pSIY+CD8+ T cells (*n* = 3). **c**, Same experimental setup as **b**, but Line-1 tumor CD4+ T cells were assessed. Pooled data from 6 independent experiments is shown for total CD4+ T cells, from 2 independent experiments for Tbet+CD4+ Th1 cells, and from 4 independent experiments for CD4+ Teffs and Tregs. **d**, Same experimental setup as **c**, but the ratios of CD8+ T cells:Tregs and CD4+ Teffs:Tregs in the tumor were assessed. Pooled data from 5 independent experiments is shown. **e**, Same experimental setup as **c-d**, but intracellular Ki-67 staining was assessed in tumors by flow cytometry. Pooled data from 3 independent experiments is shown. **b-e** One-Way ANOVA

The importance of T cells in tritherapy was determined by depleting CD8+ and/or CD4+ T cells before starting tritherapy (Fig. 3a). CD8 or CD4 depletion abrogated the tritherapy effects resulting in no mice surviving past 50 days, similar to untreated or antibody-therapy treated mice. Survival of mice depleted of both CD8+ and CD4+ T cells was decreased even further. Therefore, the tritherapy is dependent on both CD8+ and CD4+ T cells.

### Tritherapy alters the CD4+ T-cell compartment within the TME

We next assessed the effect of tritherapy on tumor T-cell infiltration. Antibody therapy increased CD8+ T-cell infiltration into tumors similar to tritherapy-treated mice (Fig. 3b). When assessing tumor-specific T cells, all therapies trended towards increased similar levels of tumor-infiltrated AH1-specific CD8+ T cells, which have led to protective antitumor responses in many BALB/c originating tumors [30, 31]. In the immunogenic Panc02-SIY tumor model, antibody therapy increased SIY-specific CD8+ T cells but the addition of peritumoral DR-BMCs did not further augment the therapy (Fig. 3b). Therefore, the percent of tumor infiltrating CD8+ T cells was unaffected by peritumoral DR-BMC inclusion compared to antibody therapy alone, suggesting the absence of cross-presentation by peritumorally administered BMCs.

The percentages of total tumor-infiltrating CD4+ T cells (Fig. 3c) also did not vary among the different treatment groups. However, further analysis showed increased Tbet+CD4+ Th1 cells and FoxP3-CD4+ effector T-cells (Teff) with decreased FoxP3 + CD4+ regulatory T-cells (Tregs) in tritherapy-treated tumors compared to all other groups (Fig. 3c), suggesting TME skewing towards antitumor immunity. Subsequently, antibody therapy increased the CD8+ T-cell:Treg ratio in the tumor without further augmentation from peritumorally administered DR-BMCs, but tritherapy significantly increased the ratio of tumor CD4+ Teffs:Tregs compared to all other groups (Fig. 3d). Similar trends were also seen in the Panc02 model (Additional file 1: Figure S5A). Therefore, GITR agonist and PD-1 blockade modestly increased the percentage of CD8+ T cells in the tumor and the addition of peritumoral DR-BMCs skewed the tumor CD4+ T-cell compartment towards an immune-stimulating response.

### Tritherapy leads to increased proliferation of CD4+ Teff cells in the tumor

Due to the variations in CD4+ Teffs and Tregs in tritherapy-treated tumors, we assessed the effect of peritumoral DR-BMCs on T-cell proliferation. Antibody therapy increased CD4+ T-cell (both Teff and Tregs) proliferation compared to untreated mice, however only tumor CD4+ Teffs and not Tregs proliferated even further with tritherapy (Fig. 3e). This increased CD4+ Teff cell proliferation was also seen in the Panc02 tumor model (Additional file 1: Figure S5B) and explains the altered Teff to Treg ratio by the tritherapy.

### Tritherapy increases functional T cells in the tumor

**Fig. 4:**
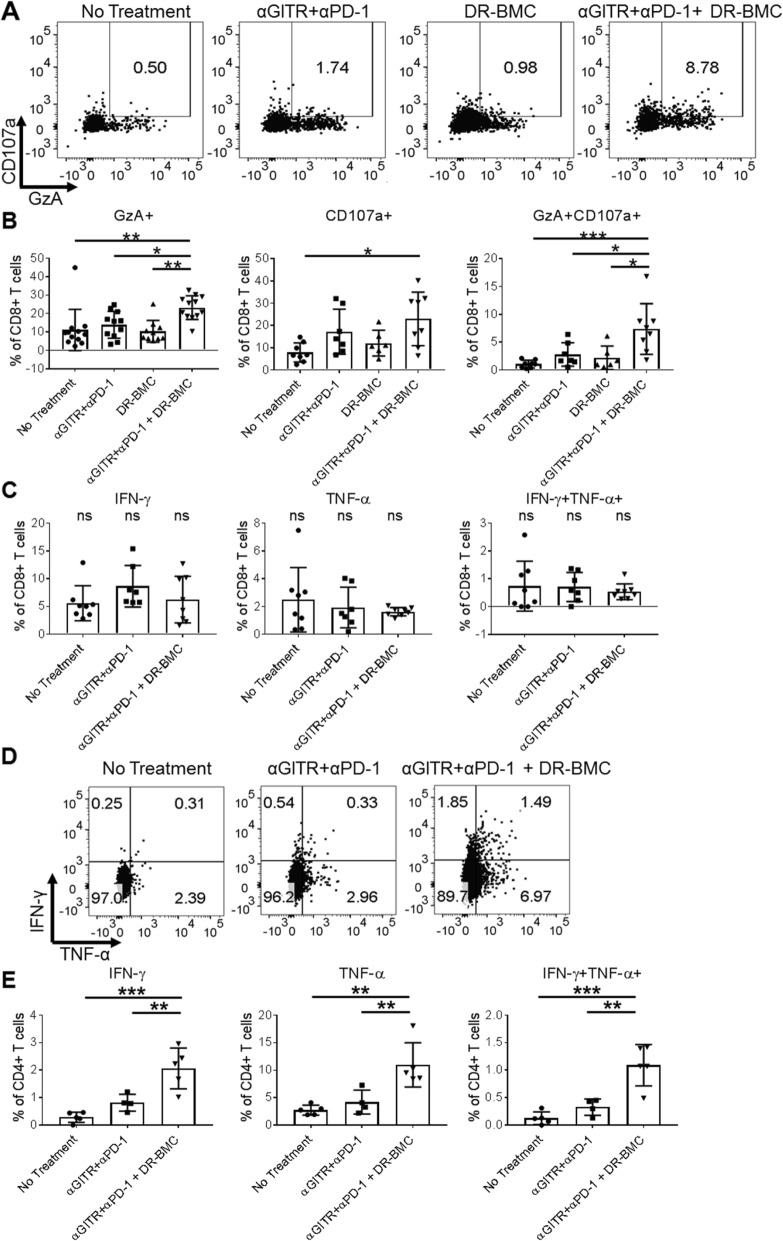
Increased effector function of CD8+ and CD4+ T cells in tumors of tritherapy-treated mice. **a**, Representative flow plots and **b**, graphical representation of intracellular GzA and surface CD107a expression on CD8+ T cells in tumors harvested 7 days after p.t. DR-BMC injections. Pooled data from 3 independent experiments is shown for GzA + CD8+ T cells, and from 2 independent experiments for CD107a + and GzA + CD107a + CD8+ T cells. One-Way ANOVA. **c**, 10 days after DR-BMC p.t. injections, Line-1 tumors were harvested 4.5 h after BFA i.v. injections and intracellular cytokine staining on CD8+ T-cells was performed. Data shown here is representative of 2 independent experiments. **d**, Same as **c** but representative flow plots of tumor-infiltrating CD4+ T cells. **e**, Same as **c-d** but cytokine production by tumor CD4+ T cells is shown. Data shown here is representative of 2 independent experiments. One-Way ANOVA

CD4+ T cells provide help to CD8+ T cells to increase their effector function [32]. Since tritherapy increased CD4+ Teffs, we next assessed the cytolytic ability of CD8+ T cells post tritherapy (Fig. 4a-b). Antibody therapy trended towards increased expression of granzyme A (GzA) and the degranulation marker, CD107a, on tumor CD8+ T cells compared to untreated mice, however a significant difference was not detected. Interestingly, tritherapy significantly enhanced GzA+, CD107a + and GzA + CD107a + CD8+ T cells (Fig. 4b). Low GzA and CD107a expression was detected in tumor CD4+ T cells with no significant differences between the groups (Additional file 1: Figure S6A). Consequently, although tritherapy did not alter CD8+ T cell tumor percentages, these cells exhibited a more cytolytic phenotype. Expression of T-cell activation markers, CD69, ICOS and TIGIT, on tumor-infiltrating T-cells was not affected (Additional file 1: Figure S6B).

Next, T-cell functional capacity was assessed by detecting in situ cytokine production 4.5 h after intravenous BFA administration. Cytokine production by tumor CD8+ T cells was not significantly different between groups (Fig. 4c). In contrast, while antibody therapy only modestly increased IFN-γ-producing CD4+ T cells in the tumor, tritherapy significantly increased IFN-γ+, TNF-α + and IFN-γ + TNF-α + CD4+ T cells compared to all other treatments (Fig. 4d-e). Taken together, these results suggest that tritherapy increases CD8+ T-cell cytolytic activity and enhances CD4+ T-cell cytokine production in the tumor.

To determine if tritherapy increased tumor-specific CD4+ T cells in the tumor, we used BALB/c Nur77GFP reporter mice. Nur77 is specifically upregulated early after T cell receptor (TCR) engagement and not as a result of inflammation [33], therefore it is a surrogate marker for antigen-specific stimulation. Mice receiving tritherapy had increased Nur77 expression on CD4+ T cells compared to untreated or antibody therapy-treated mice (Additional File 1: Figure S6C). No significant differences in Nur77 + CD8+ T cells were detected, similar to results obtained when assessing AH1 or SIY-specific CD8+ T cells (Fig. 3b). These results suggest that peritumoral administration of DR-BMCs increases tumor-reactive CD4+ T cells in the tumor. Also, antibody therapy trended towards increased TCRβ clonality within tumors, however no significant differences were detected (Additional File 1: Figure S6D). Therefore, antibody therapy led to overall T cell enrichment within the tumor but no additional change was seen with DR-BMC administration.

### Increased T-cell activation in tumor draining LNs of tritherapy-treated mice

**Fig. 5:**
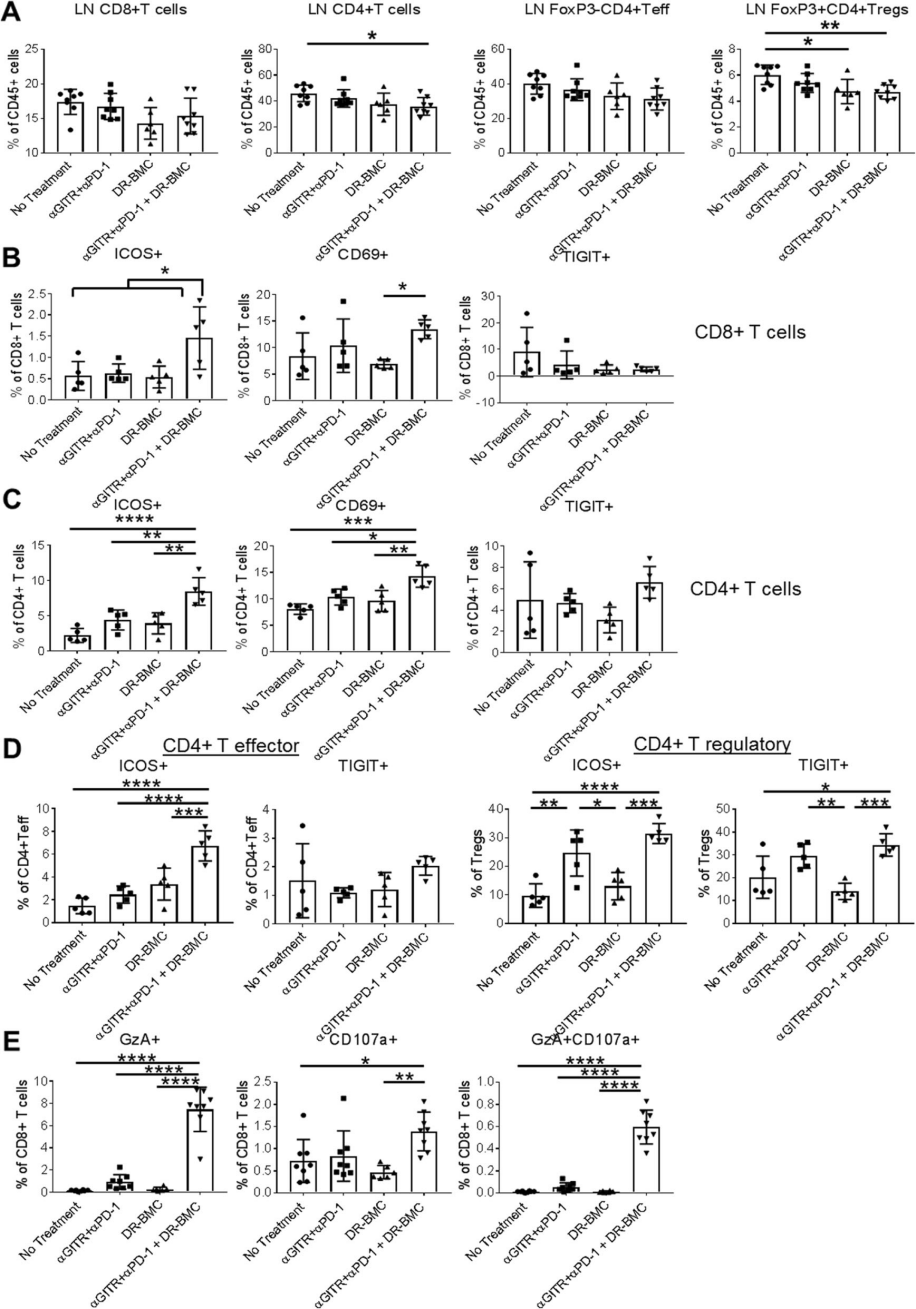
Increased T cell activation in lymph nodes of tritherapy-treated mice. **a**, LNs were harvested and analyzed by flow cytometry 7 days after p.t. DR-BMC administration. Pooled data from 2 independent experiments are shown here. **b**, Same as **a** but activation markers on CD8+ T cells and **c**, CD4+ T cells were analyzed. Shown is representative data of 4 independent experiments for ICOS expression, 2 independent experiments for CD69 expression and one experiment for TIGIT expression. **d**, Same as **a-c**, but activation markers on effector FoxP3-CD4+ T cells versus FoxP3 + CD4+ Tregs are shown. Representative data from 2 independent experiments are shown. **e**, Same as **a-d**, but cytolytic potential of CD8+ T cells from the lymph nodes are shown. Pooled data from 2 independent experiments are shown. **b-e** One-Way ANOVA

Tumor-specific T cell priming occurs in tumor draining LNs [34], therefore we assessed T-cell activation, function and proliferation in the LNs of treated mice. Similar percentages of CD8+ T cells and CD4+ Teffs were detected between groups in the LNs, however a slight decrease in total CD4+ T cells and Tregs was seen in tritherapy-treated mice (Fig. 5a). In contrast, similar T-cell percentages were seen in the spleens except that antibody therapy alone increased splenic Tregs (Additional file 1: Figure S7A).

The addition of peritumoral DR-BMCs to systemic antibody therapy led to increased ICOS and CD69 expression on both CD8+ (Fig. 5b) and CD4+ T cells (Fig. 5c) within LNs. TIGIT expression remained unaffected. ICOS is highly expressed on Tregs, therefore we further investigated effects on CD4+ T-cell subtypes. Antibody therapy increased ICOS and TIGIT expression on Tregs, however only tritherapy-treated mice had increased ICOS expression on CD4+ Teff cells in the LNs (Fig. 5d). Additionally, cytolytic activity (GzA and CD107a expression) was dramatically enhanced on CD8+ T cells within LNs of tritherapy-treated mice (Fig. 5e). Conversely, in the spleen, antibody therapy increased CD4+ T-cell activation whereas peritumoral DR-BMC inclusion did not further augment this effect (Additional file 1: Figure S7B). These results suggest that outside the tumor, systemic GITR agonist and PD-1 blockade increases peripheral T-cell activation in the spleen and LN, whereas the inclusion of peritumoral DR-BMC administration further augments the activation of CD8+ T cells and CD4+ Teffs only in tumor-draining LNs.

### Peritumoral DR-BMC administration promotes tumor rejection locally within the TME

**Fig. 6:**
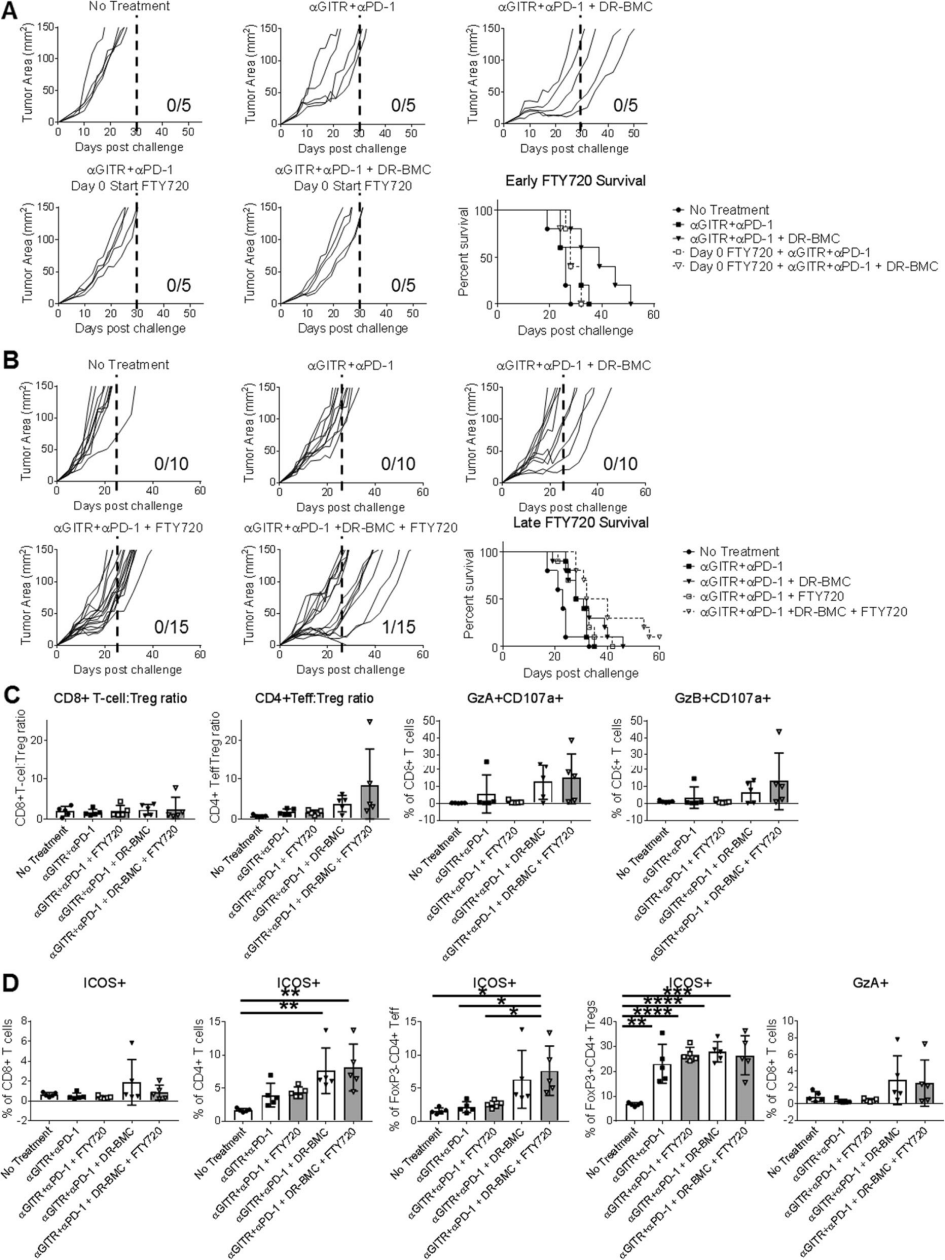
Tritherapy promotes tumor regression locally within the TME after initial lymph node T cell priming. **a**, Individual tumor growth curves and survival of antibody therapy and tritherapy-treated mice with and without daily FTY720 administered starting at day 0 before tumor inoculation. Shown is data from one independent experiment (*n* = 5). **b**, Individual tumor growth curves and survival of antibody therapy and tritherapy-treated mice with and without daily FTY720 administered starting 1 day before p.t. DR-BMC vaccination. Pooled data from 3 independent experiments are shown. **c**, Mice were treated as in **b** but sacrificed 7 days after peritumoral BMC vaccination. Line-1 tumors were harvested and analyzed by flow cytometry. **d**, Mice were treated as in **b-c** but LNs were harvested and analyzed by flow cytometry. **c**-**d** data shown is mean ± SD of one experiment with *n* = 5. One-Way ANOVA

Given the increased effector T-cell activity seen in the tumor as well as the LNs of tritherapy-treated mice, we next assessed if the LN T-cell activation was necessary for tumor rejection. For these studies, we injected the drug FTY720, a S1P1R agonist that prevents T-cell egress from secondary lymphoid structures, daily either starting before tumor inoculation or 1 day before peritumoral BMC administration. Mice that received FTY720, had decreased blood T-cell circulation during treatment (Additional file 1: Figure S8). When FTY720 was started before tumor inoculation, all tumors grew out with similar rapid kinetics (Fig. 6a), suggesting that the LNs were important for initial T-cell priming.

However, the delayed tumor growth curve seen with tritherapy was unaffected by FTY720 administration starting 1 day before peritumoral BMC administration, in which tumor regression was seen in 6.7% of mice (Fig. 6b). No difference in tumor growth was seen with antibody therapy given with or without FTY720, suggesting that the drug itself did not affect tumor growth. Moreover, tritherapy-treated mice with or without FTY720 treatment before peritumoral BMC administration showed similar increases in CD4+ Teff:Treg ratios and cytolytic CD8+ T cells in the tumors (Fig. 6c), as well as increased T-cell activation and cytolytic CD8+ T cells within the LNs (Fig. 6d). Overall, these results suggest that although T-cell activation was seen in LNs of tritherapy-treated mice, tumor rejection induced upon peritumoral DR-BMC administration was locally induced within the tumor and independent of T-cell recruitment from the LNs. However, an initial T cell priming event within the LNs before beginning antibody therapy was required in order for the peritumoral DR-BMCs to augment the effector function of the primed T cells.

### Peritumoral cross-presenting DC administration delays survival of antibody therapy-treated mice

Increased abundance of intratumoral cross-presenting DCs correlates with improved overall survival [35] by attracting [36], stimulating and expanding tumor-specific T cells [37]. These DCs are characterized by CD103 and Clec9a expression in mice. DRibbles express Clec9a ligands aiding in cross-presentation [21]. BMCs contain very low levels of CD103+ DCs and the presence of these DCs within the TME are sparse [37, 38]. Therefore, we assessed the administration of CD103+ DCs peritumorally with antibody therapy.

Generation of cross-presenting DCs from bone marrow cells led to ~ 57% MHC II + CD11c + DCs in culture with 74.7% expressing CD103 and only 23.1% expressing CD11b (Additional file 1: Figure S9A). Clec9a was expressed on 32.4% of the DCs and very few cells expressed MDSC (CD11b + GR1+) or macrophage (CD24-F4/80+) markers. Upon peritumoral DR-DC injections with antibody therapy (DR-DC-tritherapy), 20% of tumors regressed, similar to mice receiving DR-BMC-tritherapy. DR-DC-tritherapy mice showed marginal delays in tumor growth with a median survival of 46 days as compared to 37.5 days seen with DR-BMC-tritherapy (Additional file 1: Figure S9B). In addition, the injected DCs remained in the tumors and were not detected in LNs or spleens (Additional file 1: Figure S9C) similar to that seen with BMCs, suggesting that the injected DCs also orchestrate the local immune stimulation occurring within tumors.

### Antigen presentation in situ is not required by peritumoral DR-BMC/DC administration for tritherapy efficacy

**Fig. 7:**
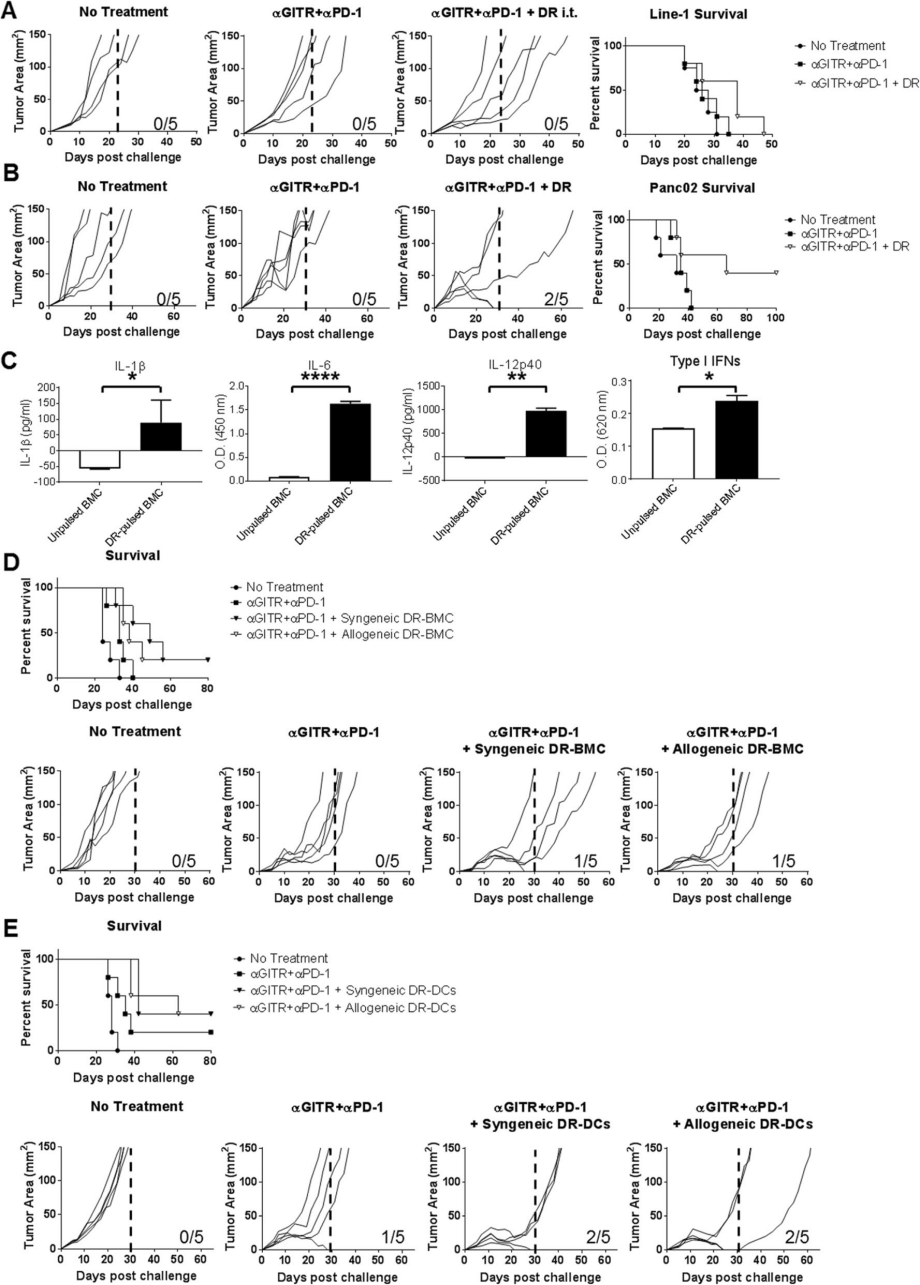
Tritherapy efficacy is independent of antigen presentation by peritumorally administered APCs. **a**, Line-1-tumor bearing mice were treated i.p. with anti-GITR antibody on days 5 and 8 and anti-PD-1 antibody on days 10, 12, and 14. Line-1 cell derived DRibbles were peritumorally administered on day 12. Individual tumor growths and overall survival is shown. Representative data from 1 experiment is shown (*n* = 5). **b**, Panc02-tumor bearing mice were treated as in **a** except DRibbles were derived from the Panc02 cell line. Representative data from 1 experiment is shown (*n* = 5). **c**, Washed unpulsed and DRibbles-pulsed day 8 BMCs were cultured for 24 h after which the supernatant was collected and analyzed by ELISA for IL-1beta, IL-6 or IL-12p40. Type I IFN presence in the supernatant was analyzed using B16Blue-IFNa/b cells. Data (mean ± SD) from one independent experiment performed in triplicate wells for IL-1β and IL-6 or duplicate wells for IL-12p40 and Type I IFNs is shown. **d**, Line-1 tumor bearing BALB/c mice were treated with BMC-tritherapy using BMCs derived from either syngeneic BALB/c mice or allogeneic C57BL/6 mice bone marrows. BMCs were pulsed with Line-1 cell-derived DRibbles before peritumoral administration. Representative data from 1 experiment is shown (n = 5). **e**, Same as **d** however mice were treated with syngeneic BALB/c or allogeneic C57BL/6 CD103+ DCs pulsed with DRibbles derived from Line-1 cells. Representative data from 1 experiment is shown (n = 5)

DRibbles contain tumor antigens and activate an innate inflammatory response [21, 25]. We assessed if DRibbles could replace DRibbles-pulsed-BMCs in the tritherapy. DR-tritherapy led to enhanced survival in both the Line-1 and Panc02 tumor models compared to antibody therapy alone (Fig. 7a-b). Mice in the Line-1 tumor model were not cured unlike those treated with DR-BMC-tritherapy or DR-DC-tritherapy. In the Panc02 tumor model, DR-tritherapy-treated mice showed similar cure rates compared to those receiving DR-BMC-tritherapy (Fig. 7c).

Pulsing BMCs with DRibbles increased IL-1β, IL-6, IL-12p40 and Type I IFN production compared to unpulsed BMCs (Fig. 7c). Therefore, besides providing antigen, DRibbles can generate an innate inflammatory response. We previously saw better tritherapy efficacy when DR-BMCs were administered later at day 12 after T-cell priming had most likely already occurred (Additional file 1: Figure S1). Therefore, the necessity of T-cell priming by the transferred BMCs/DCs was assessed by using allogeneic BMCs/DCs containing mismatched MHC molecules thus rendering them unable to present antigen to host T cells. Mice receiving tritherapy with allogenic DR-pulsed-BMCs/DCs led to a similar increase in survival as mice receiving tritherapy with syngeneic DR-pulsed-BMCs/DCs (Fig. 7d-e). These results suggest that peritumorally administered DR-pulsed-APCs do not need to present antigens in situ for tritherapy efficacy and that perhaps a generated inflammatory response could be responsible for the enhanced tumor regression.

## Discussion

In this present study, the efficacy of systemically administered GITR agonist and PD-1 blockade was augmented by peritumoral DR-pulsed-BMC/DC administration independently of antigen presentation and through local alterations of T-cell effector functions within the tumor. Herein, we found that peritumorally-administered BMCs/DCs remained within the tumor and did not migrate to LNs as is expected of activated DCs. FTY720 studies suggested that tumor regression in tritherapy-treated mice was induced by a local influence of the peritumoral DR-BMCs on T cells within the tumor and did not require the effects of the LN even though robust T-cell activation was detected in LNs. In addition, DR-BMCs administered a week after beginning antibody therapy led to better antitumor responses compared to earlier administration, and the use of MHC-mismatched APCs in the tritherapy led to similar results as MHC-matched APCs. Together, these results demonstrate that antigen presentation by peritumorally-injected-BMCs is not necessary for tritherapy efficacy. Despite this, the inclusion of intratumoral DRibbles-pulsed APCs to antibody therapy promoted the further expansion and differentiation into Teff cells and heightened the cytolytic potential of CD8+ T cells in the tumor.

Pulsing BMCs with DRibbles increased the in vitro production of pro-inflammatory cytokines IL-6, IL-1β, IL-12 and Type I IFNs and replacing DR-pulsed-BMCs with DRibbles also delayed tumor growth kinetics, albeit not as prominently. These results suggest that a local inflammatory response mediated by DRibbles pulsing could lead to the sustained antitumor effects seen. Inflammatory cytokines, such as IL-12 and Type I IFNs, have shown to increase proliferation, adhesion and costimulatory molecule expression, activation, effector function of effector and memory T cells, [39] and to lower TCR antigen sensitivity required for activation [40]. In addition, the inflammatory cytokine milieu can also effect T-cell recruitment by altering sensitivity towards selectins [39], increasing tumor vasculature as evidenced by increased IL-6 production [41] and by inducing T-cell chemoattractants such as CCL5 and CXCL9 [42, 43]. Intratumoral administration of oncolytic viruses that promoted inflammatory cytokine production, in particular Type I IFNs, have also led to similar enhancements of systemic CTLA-4 blockade in which antitumor T-cell responses in distant tumors were also seen [44]. Therefore, altering the inflammatory cytokine milieu can positively impact local effector and memory T cells and sustain T-cell immunity within the tumor.

Since antigen presentation by peritumorally-transferred-DR-APCs was not required and mice receiving DRibbles-tritherapy demonstrated enhanced survival, it is possible that administrating DAMPs alone that initiate an innate inflammatory response may be sufficient to enhance the effects of antibody therapy. Future studies identifying the necessity of individual inflammatory mediators or DAMPs sufficient to enhance the effects of antibody therapy will prove beneficial. However, considering the vast number of inflammatory mediators activated by DRibbles, it is highly possible that a combination of many DAMPs will be required to mediate the same antitumor effects seen with DRibbles.

Previous studies using GITR agonist and PD-1 blockade show marginal synergy between the two antibodies with minimal tumor clearance, therefore, combination with chemotherapy, vaccination or radiation to further prime an immune response was assessed to increase tumor clearance [2, 4, 16]. These studies differ from ours in which peritumorally-administered DR-BMCs were used to safely manipulate the T-cell response generated previously by antibody therapy and not to necessarily prime more T-cells. A similar study demonstrated that intratumoral or systemic GITR agonist antibody combined with intratumoral administration of DCs and CD4+ T cells led to enhanced survival compared to subcutaneous DCs and intravenous CD4+ T cells [20]. The authors attributed intratumoral delivery with increased antitumor and decreased pro-tumor cytokines/chemokines within the TME which in turn increased the tumor influx of CD8+ T cells, also suggestive of how locally modulating the inflammatory milieu, in this case with tumor lysate-pulsed DCs, can recruit T-cells to the tumor. Consequently, intra/peritumoral DC administrations are more beneficial than the traditional subcutaneous administration route in modulating the TME locally towards an antitumor environment and could potentially be combined with many different agents that previously prime T-cell immunity but are insufficient in leading to tumor regression. Given that DC-tritherapy led to similar results as BMC-tritherapy, a clinically relevant and safe approach would be to isolate natural circulating DCs or monocytes from patient peripheral blood mononuclear cells (PBMCs) via apheresis. Since APC antigen presentation was not required, allogeneic DCs could also be used.

Previous studies demonstrate that cross-presenting DC presence within the TME suggests better synergy with T-cell targeted therapies. Early i.p. Flt3L and intratumoral polyIC administrations synergized with PD-L1 blockade [38] or TNFR CD137 agonist and PD-1 blockade combination therapy [45] through the expansion of CD103+ DCs within the TME. These studies suggested that tumor-resident cross-presenting DCs were important for T-cell tumor infiltration allowing for further manipulation by T-cell targeted therapies. In our study, BMCs had very low levels of CD103 or IRF8, and major manipulations of CD8+ T cells within the TME were not detected, suggesting that the majority of BMCs were not cross-presenting DCs. Interestingly, peritumoral injections of higher percentages of cross-presenting DCs expressing CD103 and CLEC9A did not significantly improve cure rates when used in the tritherapy, although delayed median survival was seen. Consistent with our data showing that antigen presentation by the injected APCs was not necessary for tritherapy efficacy, a recent study highlights the ability of Batf3-dependent-DCs to lead to tumor rejection by methods other than cross-presentation [46], which may play a role in the tritherapy. Therefore, we predict that peritumorally-administered DR-BMCs/DCs did not increase tumor T-cell infiltration or priming, but manipulated the local immune TME that was previously established by GITR agonist treatment, perhaps through an antitumor inflammatory response involving cytokine/chemokine production by the DRibbles-activated APCs.

Tritherapy led to increased survival of mice with 20% cures compared to antibody therapy alone, in which all mice succumbed to tumor burden. However, many tumors began to regress with tritherapy but would then progress about a week after BMC/DC administration, whereas some were completely refractory to treatment, analogous to what is seen in patients. Live injected BMCs/DCs remained within the tumor for at least 2 weeks after peritumoral administration and multiple follow up peritumoral DR-BMC administrations did not improve efficacy (data not shown), suggesting that the cells are still present and viable to exert effects even when some tumors begin to progress. Additionally, peritumorally administered IL-2 or IL-15 did not further potentiate tritherapy efficacy (data not shown). It is possible that tumor escape mechanisms could be at play in which tumor cells may lose neoantigens or pursue immune evasion tactics. Also, GITR agonist therapy alone may not generate enough tumor-specific T-cells thus, the addition of early vaccination or radiation to expand tumor-specific T cells may provide benefit to the therapy. Another possibility is that despite PD-1 blockade, prolonged exposure of T cells to the TME could lead to dysfunction/exhaustion thus allowing tumors to progress after an initial regression. Therefore, additional checkpoint inhibitors, such as CTLA-4, TIM-3, LAG-3, etc., could be assessed in combination.

## Conclusions

DC vaccines administered systemically in the clinic failed in leading to tumor regression [47, 48], however peritumoral administration may prove more beneficial. We report that peritumoral administration of DRibbles-pulsed-APCs can enhance the efficacy of systemic T-cell-targeted immunotherapies by locally manipulating the TME. Enhanced efficacy was seen even in the absence of antigen presentation and perhaps through local innate inflammatory modulations mediated by DRibbles-pulsed-APCs, thus creating a supportive environment in which T-cell immunity is sustained.

## Supplementary information

**Additional file 1: Table S1.** List of antibodies used for flow cytometry analysis. **Figure S1.** Timing assessment of peritumoral DR-BMC administration. **Figure S2.** Flow cytometry analysis of DRibbles-pulsed BMCs before vaccination. **Figure S3.** Flow cytometry analysis of peritumorally injected BMCs in tumors of tritherapy-treated mice 7 days after peritumoral BMC administration. **Figure S4.** Tritherapy cured mice are protected from tumor rechallenge. **Figure S5.** Tritherapy alters the tumor CD4+ T-cell compartment in the Panc02 model. **Figure S6.** Similar activation marker expression but increased tumor-specific CD4+ T cells in tumors of tritherapy-treated mice. **Figure S7.** Antibody therapy alters peripheral CD4 + T cells. **Figure S8**. FTY720 decreases circulation of T cells in the blood. **Figure S9.** DC-tritherapy similarly delays tumor growth.

## Data Availability

All data generated or analyzed during this study are included in this published article and its supplementary information files.

## Abbreviations

Ab: Antibody
APCs: Antigen-presenting cells
BFA: Brefeldin A
BMCs: Bone marrow cells
CM: Complete media
CTLA-4: Cytotoxic T-lymphocyte-associated antigen 4
DCs: Dendritic cells
DR: DRibbles
FBS: Fetal bovine serum
GITR: Glucocorticoid-induced TNFR-related protein
GzA: Granzyme A
i.p: Intraperitoneally
i.v: Intravenously
IACUC: Institutional Animal Care and Use Committee
LNs: Lymph nodes
MDSCs: Myeloid derived suppressor cells
p.t: peritumorally
PBMCs: Peripheral blood mononuclear cells
PD-1: Programmed cell death protein 1
Teff: effector T cells
TME: Tumor microenvironment
TNF: Tumor necrosis factor
TNFR: TNF receptor
Tregs: regulatory T cells
