# Supplementary material for: Peritumoral administration of DRibbles-pulsed antigen-presenting cells enhances the antitumor efficacy of anti-GITR and anti-PD-1 antibodies via an antigen presenting independent mechanism
Source: J Immunother Cancer. 2019 Nov 20;7:311. doi: 10.1186/s40425-019-0786-7 (PMC6865022; doi:10.1186/s40425-019-0786-7)
Supplement: Supplementary file 1 — Additional file 1: Table S1. List of antibodies used for flow cytometry analysis. Figure S1. Timing assessment of peritumoral DR-BMC administration. Figure S2. Flow cytometry analysis of DRibbles-pulsed BMCs before vaccination. Figure S3. Flow cytometry analysis of peritumorally injected BMCs in tumors of tritherapy-treated mice 7 days after peritumoral BMC administration. Figure S4. Tritherapy cured mice are protected from tumor rechallenge. Figure S5. Tritherapy alters the tumor CD4+ T-cell compartment in the Panc02 model. Figure S6. Similar activation marker expression but increased tumor-specific CD4+ T cells in tumors of tritherapy-treated mice. Figure S7. Antibody therapy alters peripheral CD4 + T cells. Figure S8. FTY720 decreases circulation of T cells in the blood. Figure S9. DC-tritherapy similarly delays tumor growth. [file 40425_2019_786_MOESM1_ESM.pdf]

Additional File 1

Supplementary Materials and Methods:

Preparation of DRibbles-pulsed cells:

DRibbles concentration was assessed using the BCA Protein Assay (Thermo Scientific) kit. To generate DRibbles-pulsed BMCs or DRibbles-pulsed DCs, $5x10^6$ cells/ml were cultured with 30 µg/ml DRibbles for 4.5 hr at 37˚C in a 50-ml conicle. Cells were then washed with PBS before use. To assess cell location after peritumoral injections, DR-pulsed BMCs/DCs were stained with PKH67 Green (Sigma) or CellVue Claret Far Red fluorescent dye (Sigma) according to manufacture instructions beforehand. Briefly, a mixture of 1 ml of Diluent C and 4 µL dye were added to 2×10^7 cells resuspended in 1 ml of Diluent C for 4 min before quenching with FBS. Cells were washed 3x with media followed by PBS before injecting.

Tumor challenge and treatment: To deplete CD8+ and CD4+ cells, 100 µg anti-CD8 Ab and 200 µg anti-CD4 Ab were administered i.p. respectively, on day 4. Anti-CD4 and anti-CD8 antibodies led to over 99.5% depletion of respective T cells from mouse spleens after 24 h of administration. FTY720 (Cayman Chemical Company) was dissolved in DMSO to 20 mg/ml, aliquoted and stored at -20˚C. Daily, an FTY720 aliquot was diluted in PBS to 200 µg/ml before injecting 100 µl i.p.

Intracellular flow cytometry staining: Cells were incubated with bovine IgG to block Fc receptors at 4˚C for 30 min before surface staining. Cells were fixed and permeabilized using Foxp3/transcription factor staining buffer kit (eBioscience) overnight at 4˚C or for 30 min at

room temperature, and then washed and stained intracellularly in permeabilization buffer. The antibodies used for flow cytometry staining are depicted in Supplementary Table S1.

To study *in situ* cytokine production, 5 h before mice were euthanized, 250 μg brefeldin A (BFA; Thermo Fisher Scientific) was injected intravenously (i.v.). Tumors, LNs and spleens were harvested and dissociated in the presence of 10 μg/ml BFA. Flow cytometry staining occurred in the presence of BFA until cells were fixed and permeabilized.

TCR Sequencing

Line-1 tumors were harvested 6 days post p.t. DR-BMC injections. The DNeasy blood and tissue kit (Qiagen) was used to extract total genomic DNA. The Mouse ImmunoSEQ kit from Adaptive Biotechnologies was used for survey sequencing of the TCRβ chain.

Supplementary Table S1: List of antibodies used for flow cytometry analysis.

Antibody	Company	Catolog Number
Fixable Viability Stain 700	BD Biosciences	564997
BV711-labeled anti-CD45	BD Biosciences	563709
APC-Cy7-labeled anti-CD45	BD Biosciences	557659
BV510-labeled anti-CD3e	BD Biosciences	563024
BV786-labeled anti-CD8a	BD Biosciences	563332
BV605-labeled anti-CD4	BD Biosciences	563151
APC-labeled AH1 Tetramer	MBL International	TB-M521-2
PE-labeled anti-pSIY	ProImmune	F2B-D
BV650-labeled anti-Tbet	BD Biosciences	564142
PE-labeled anti-FoxP3	eBioscience	12-5773-82
PE-Cy7-labeled anti-Ki-67	Thermo Fisher Scientific	25-5698-80
APC-labeled anti-Granzyme A	eBioscience	17-5831-82
PerCP-eFl710-labeled anti-CD107a	eBioscience	46-1071-82
PE-labeled anti-Granzyme B	Thermo Fisher Scientific	MHGB04
PE-labeled anti-IFN-ɣ	BD Biosciences	554412
BV421-labeled anti-IFN-ɣ	BD Biosciences	563376
BV510-labeled anti-TNF-α	BD Biosciences	563386
AF647-labeled anti-ICOS	BD Biosciences	563469
PE-labeled anti-CD69	BD Biosciences	557392
BV421-labeled anti-TIGIT	BD Biosciences	565270
BV605-labeled anti-I-A/I-E	BD Biosciences	563413
BV786-labeled anti-CD11c	BD Biosciences	563735
BV480-labeled anti-CD11b	BD Biosciences	566149
FITC-labeled anti-Ly-6G and Ly-6C (GR-1)	eBioscience	553127
BV421-labeled anti-CD103	BD Biosciences	562771
PE-labeled anti-Clec9a	BioLegend	143504
PE-eFluor610-labeled anti-F4/80	Thermo Fisher Scientific	61-4801-82
FITC-labeled anti-F4/80	Thermo Fisher Scientific	11-4801-82
PerCPeFl710-labeled anti-IRF8	eBioscience	46-9852-82
PE-labeled anti-CCR7	eBioscience	12-1971-82
BV711-labeled anti-PD-L1	BD Biosciences	563369
PE-Cy7-labeled anti-CD24	Thermo Fisher Scientific	25-0242-82

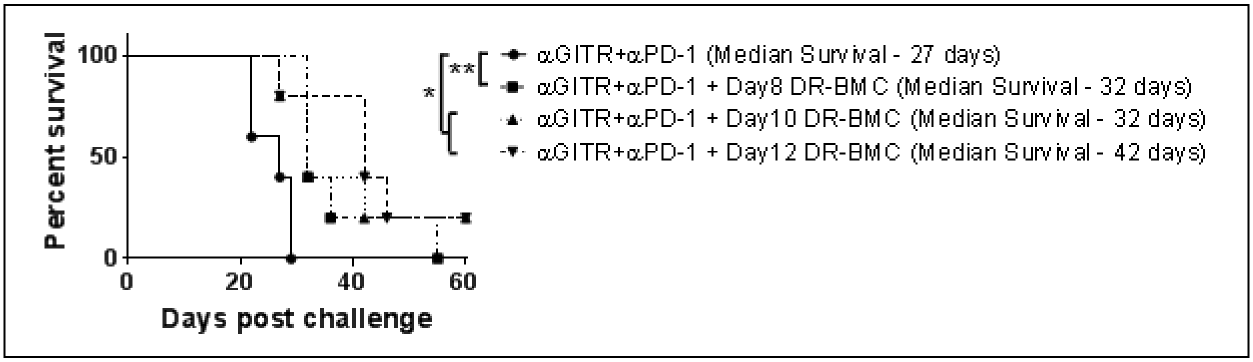

Figure S1. Timing assessment of peritumoral DR-BMC administration. Line-1-tumor bearing mice were treated i.p. with anti-GITR antibody on days 5 and 8 and anti-PD-1 antibody on days 10, 12, and 14. DR-pulsed BMCs were peritumorally administered on either day 8, 10 or 12. Overall survival is shown. Representative data from 1 experiment is shown (n = 5). Log-rank Mantel-Cox test compared tritherapy groups to antibody therapy.

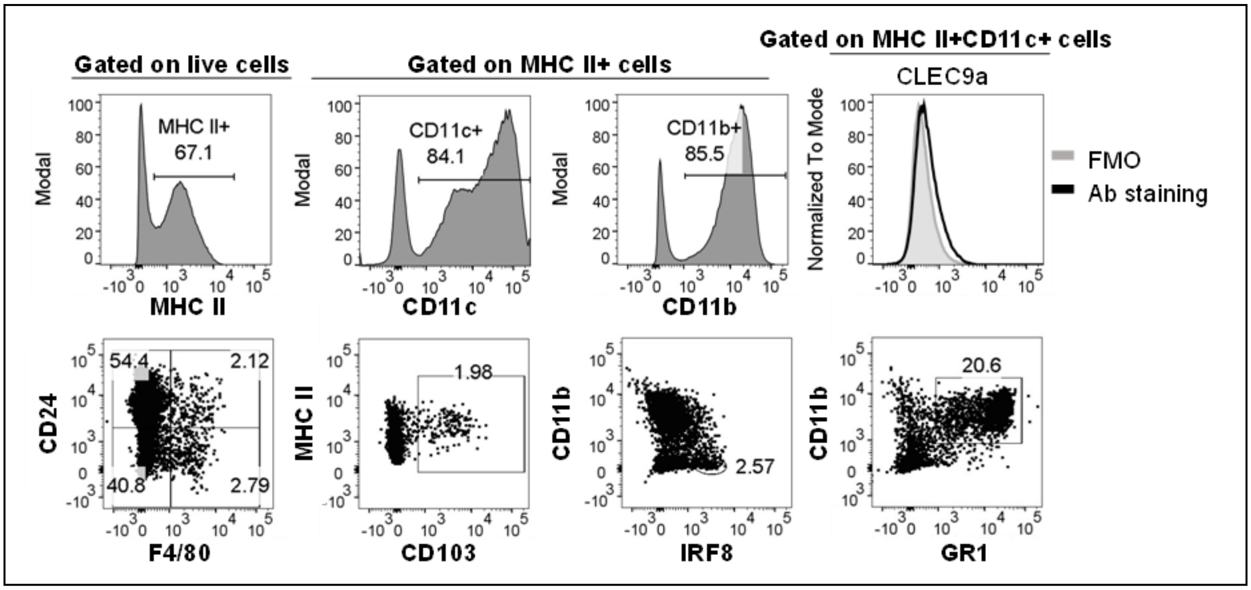

Figure S2. **Flow cytometry analysis of DRibbles-pulsed BMCs before vaccination**. **A**, Bone marrow cells were harvested and cultured in complete media for 8 days. On day 8, the resulting BMCs were harvested and pulsed with DRibbles for 4.5 h at 37˚C, 5% CO_2. The cells were washed by centrifugation and analyzed by flow cytometry. Representative data from 2 independent experiments are shown.

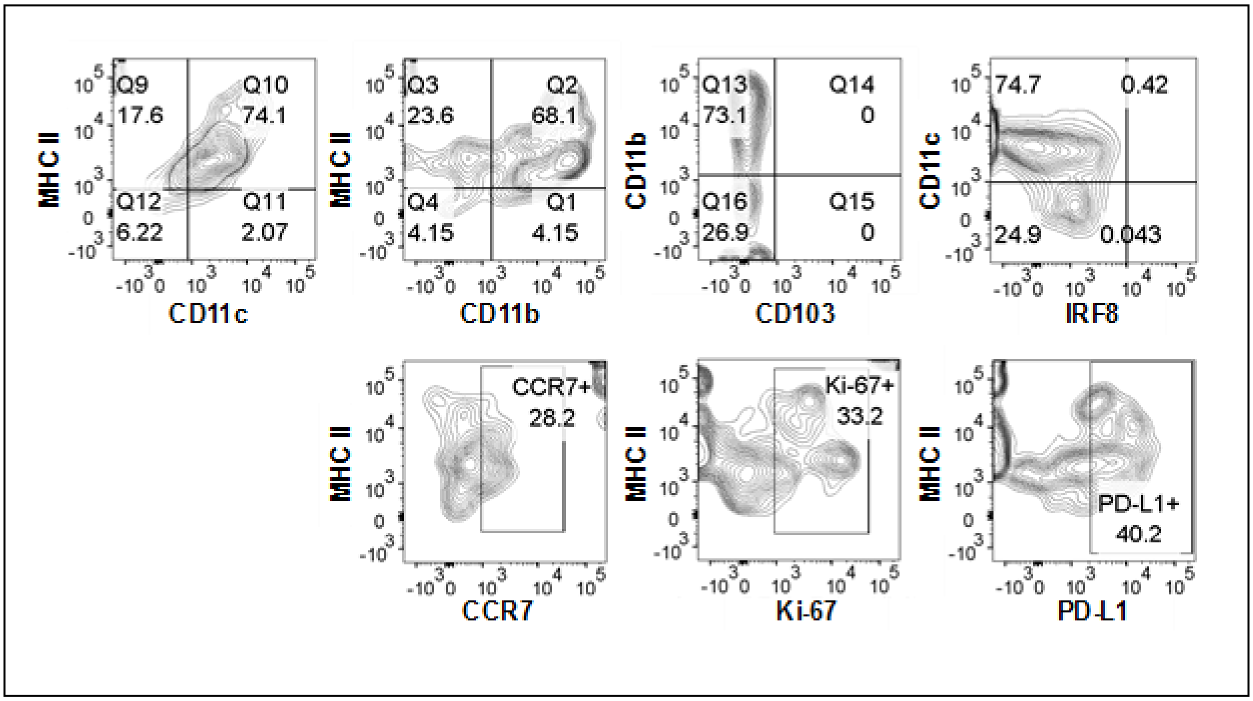

Figure S3. Flow cytometry analysis of peritumorally injected BMCs in tumors of tritherapy-treated mice 7 days after peritumoral BMC administration. Line-1 tumor bearing mice were treated with the tritherapy. DR-pulsed BMCs were labeled with PKH67 before p.t. administration. Seven days after p.t. PKH67-labeled BMC administration, tumors were harvested and PKH67+ cells were assessed by flow cytometry. The data plots on the top row are representative data from 2 independent experiments and the bottom row plots are representative data from 1 experiment (n = 4).

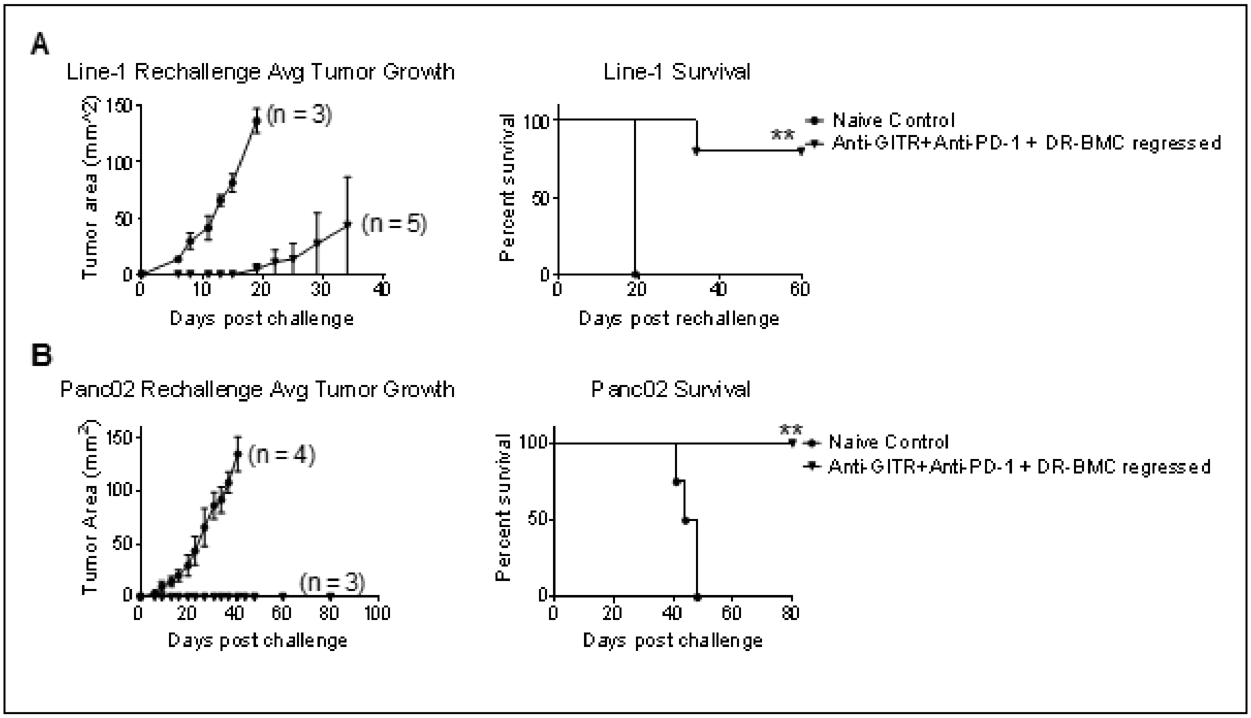

Figure S4. Tritherapy cured mice are protected from tumor rechallenge. A, Line-1 tumor-bearing mice which exhibited fully regressed tumors after tritherapy were rechallenged with Line-1 tumor cells on the opposite flank. Average tumor growth and survival was assessed. Data shown here is representative of one experiment. **B**, Panc02 tumor-bearing mice which exhibited fully regressed tumors after tritherapy were rechallenged with Panc02 tumor cells on the opposite flank. Average tumor growth and survival was assessed. Data shown here is representative of one experiment.

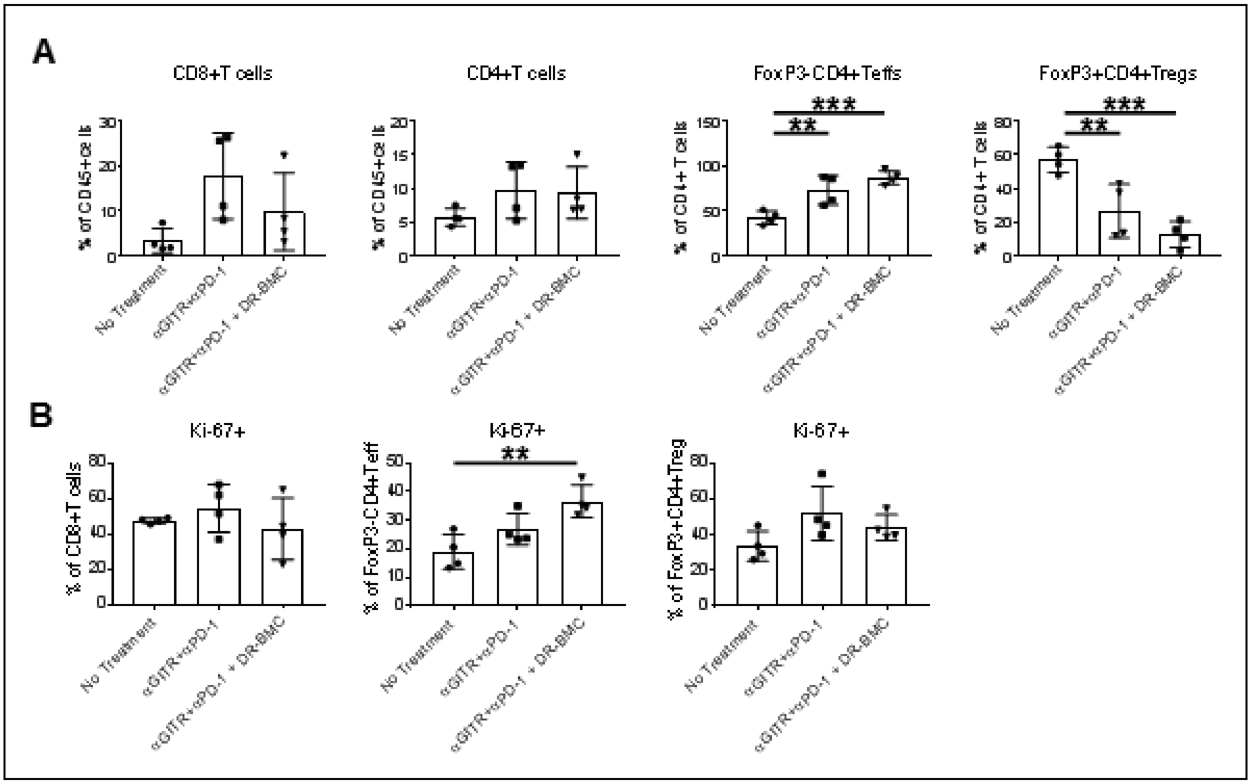

Figure S5. Tritherapy alters the tumor CD4+ T-cell compartment in the Panc02 model. A, Panc02 tumors were harvested 7 days after peritumoral BMC administration and assessed by flow cytometry for tumor-infiltrating T-cells. Data shown is mean ± SD of one experiment with n = 4. **B**, Panc02 tumors were harvested from Panc02-tumor-bearing mice 7 days after p.t. BMC vaccination and Ki-67 expression was assessed by intracellular flow cytometry analysis. Data shown is mean ± SD of one experiment with n = 4. **A-B** One-Way ANOVA.

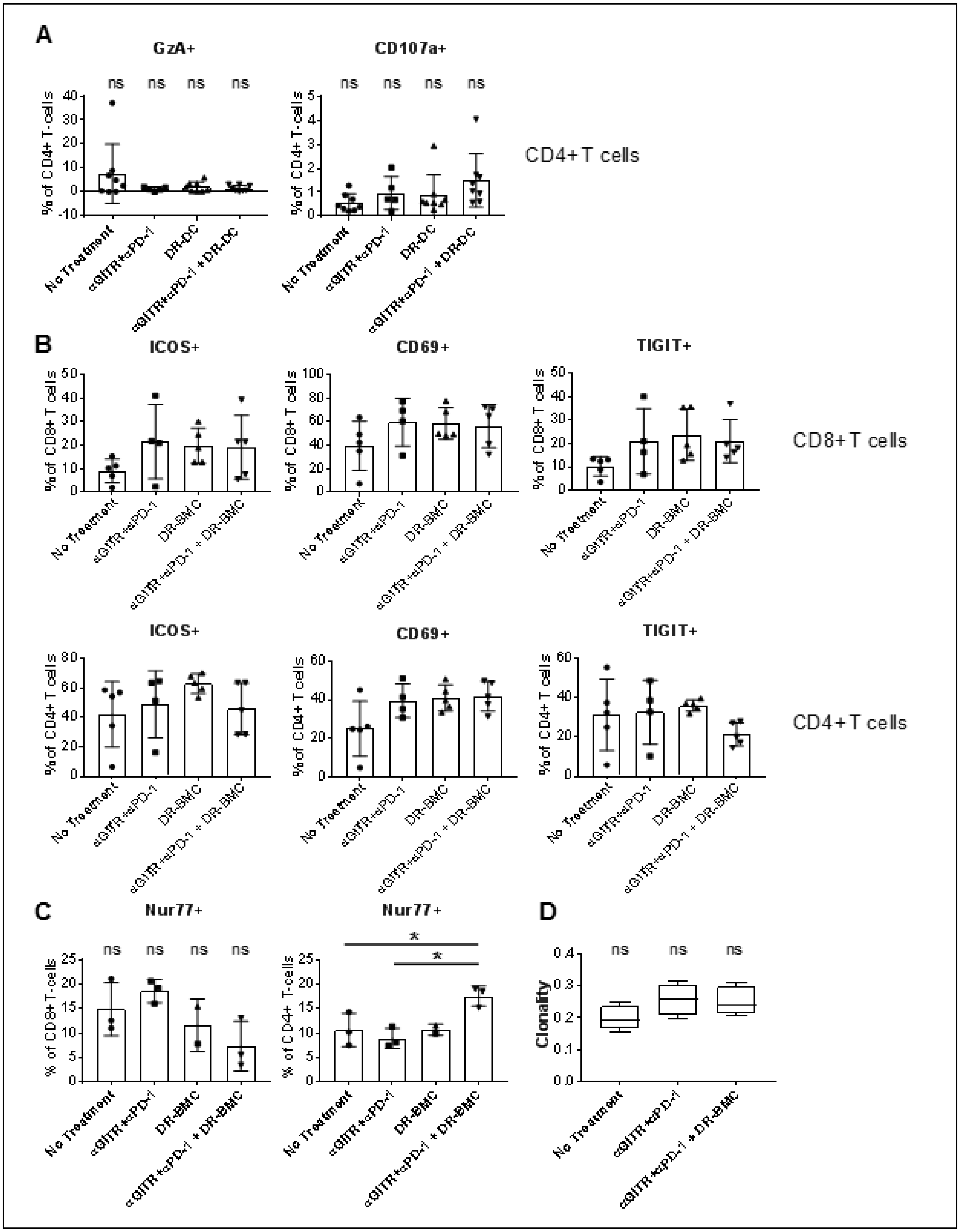

111 **Figure S6. Similar activation marker expression but increased tumor-specific CD4+ T cells**

112 **in tumors of tritherapy-treated mice. A**, Tumors were harvested 7 days after p.t. DR-BMC

injections and analyzed by flow cytometry for intracellular granzyme A and surface CD107a expression on CD4+ T cells. Pooled data (mean ± SD) from two independent experiments with n = 4 each is shown here. **B,** Same as **A**, but tumors were analyzed for activation markers. Shown here is representative data of 4 independent experiments for ICOS expression, 2 independent experiments for CD69 expression and one experiment for TIGIT expression. **C**, Line-1 tumors were harvested from BALB/c Nur77GFP mice, 3-5 days after p.t. DR-BMC injections and analyzed by flow cytometry for GFP expression. Shown here is representative data of 3 independent experiments. **D**, Line-1 tumors were harvested 6 days after p.t. DR-BMC injections. Genomic DNA was extracted and TCRβ sequencing was performed. One-Way ANOVA.

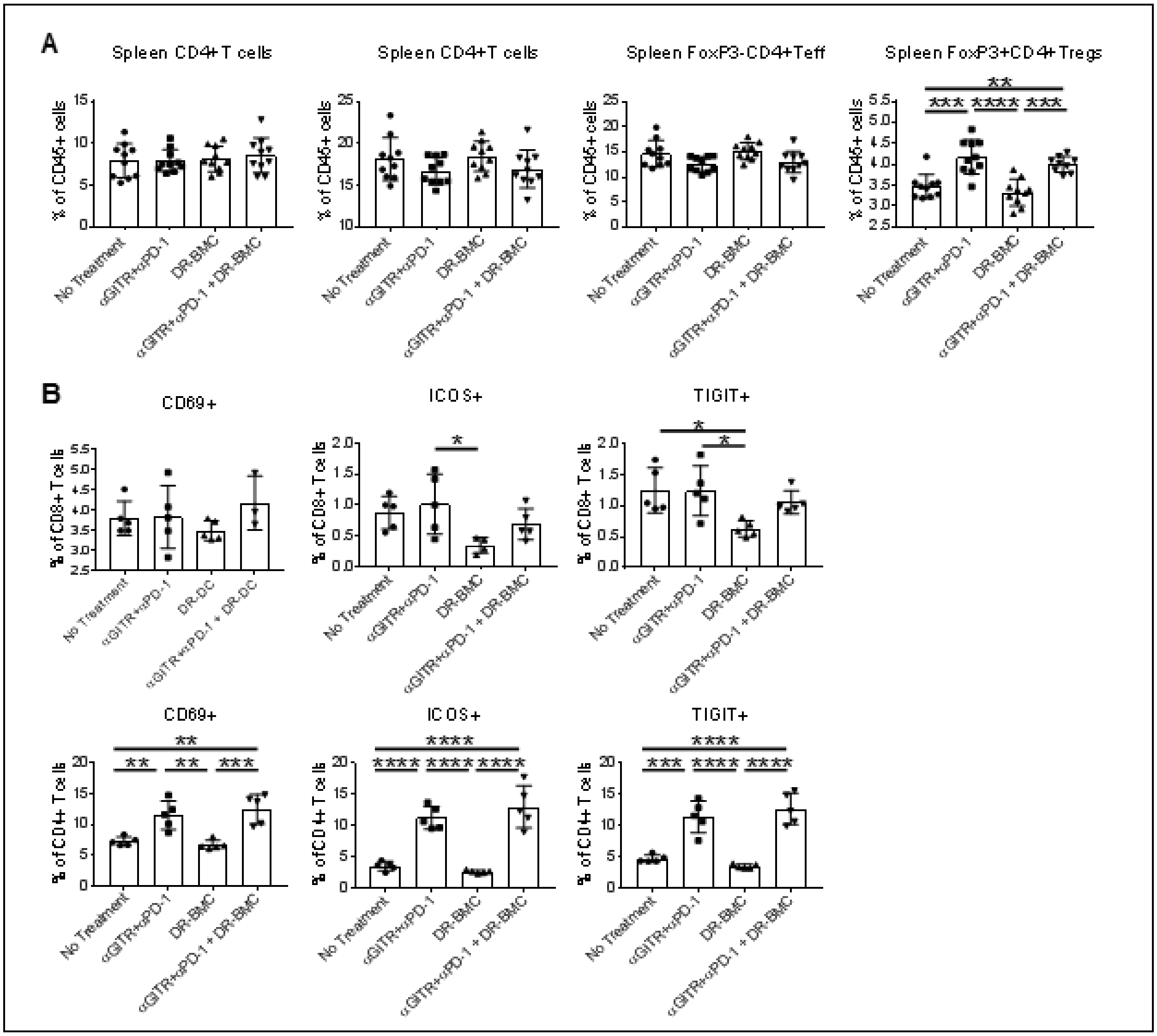

Figure S7. Antibody therapy alters peripheral CD4+T cells. **A**, 7 days after p.t. DR-BMC injections, spleens were harvested and analyzed by flow cytometry. Pooled data from 2 independent experiments are shown here. **B**, Same as **A** but spleens were analyzed by flow cytometry for activation markers on CD8+ T cells (top) and CD4+ T cells (bottom). Shown here is representative data of 4 independent experiments for ICOS expression, 2 independent experiments for CD69 expression and one experiment for TIGIT expression. **A-B** One-Way ANOVA.

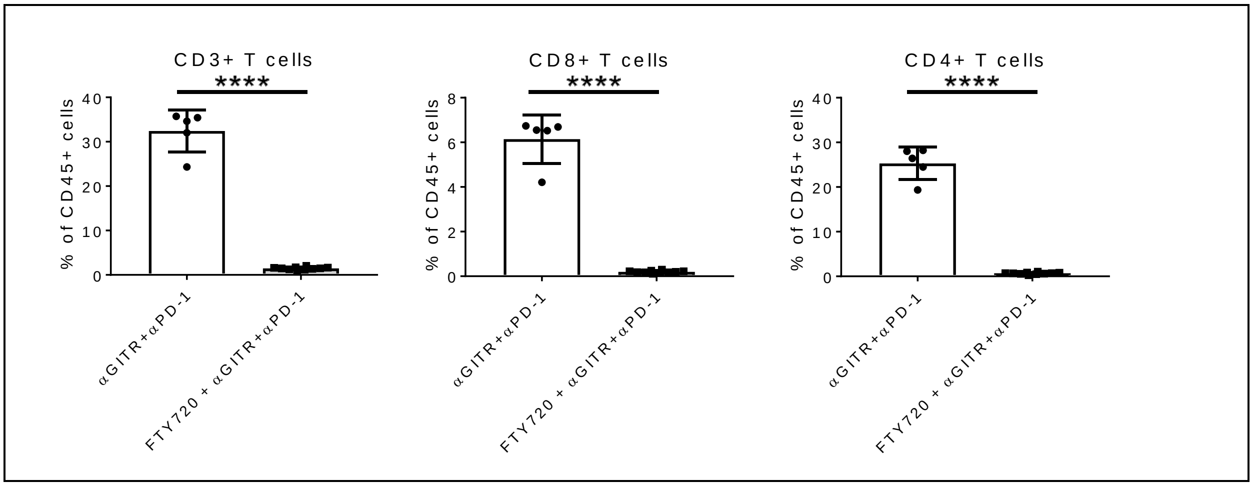

Figure S8. FTY720 decreases circulation of T cells in the blood. Blood from mice treated with antibody therapy with or without FTY720 was assessed for T cells by flow cytometry analysis on the 5th day of FTY720 administration. Representative data from 4 independent experiments are shown. Student's t-test.

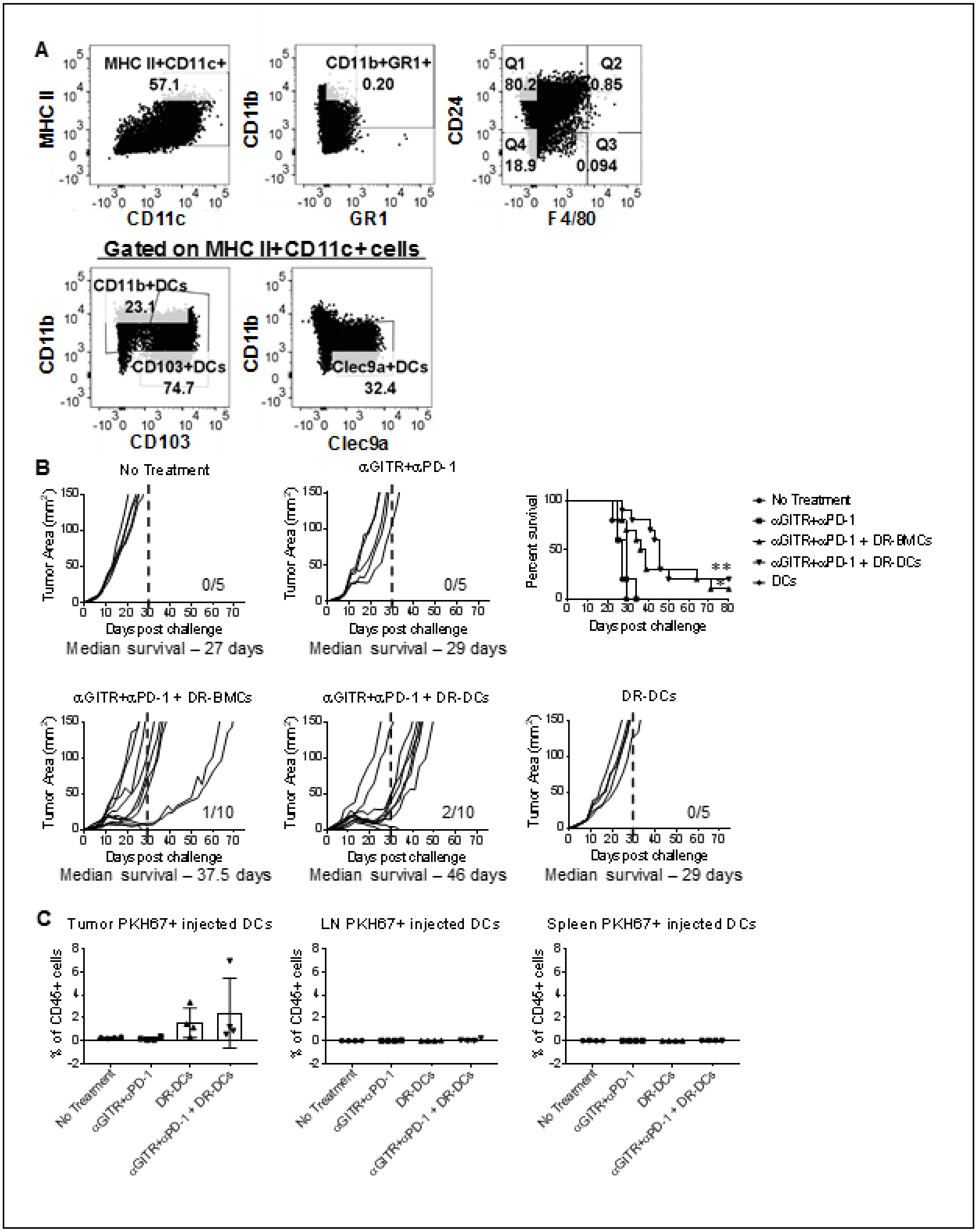

Figure S9. DC-tritherapy similarly delays tumor growth. A, Day 15 dendritic cells were harvested and pulsed with DRibbles for 4.5 h at 37˚C, 5% CO_2. The cells were washed by

centrifugation and analyzed by flow cytometry. Representative data from 3 independent experiments are shown. **B**, Mice were inoculated with $2x10^5$ Line-1 cells s.c. on day 0 followed by 200 µg of anti-GITR Ab on day 4 and 7, and 200 µg of anti-PD-1 Ab on day 9, 11 and 13. Mice were peritumorally injected with $2x10^6$ DR-pulsed BMCs or DR-pulsed DCs on day 11. Individual tumor growth curves and overall survival are shown. Representative data from 2 independent experiments are shown. Log-rank Mantel-Cox test compared BMC-tritherapy or DC-tritherapy to antibody therapy. **C**, PKH67-labeled cells in the tumor, LN or spleen of mice 7 days after peritumoral injection of PKH67-labeled DCs. Representative data (mean ± SD) with n = 4 from one independent experiment is shown.
